# Metabolic Dysfunction and T-Cell Exhaustion in Colorectal Cancer: Mechanisms, Microenvironment Remodeling, and Therapeutic Opportunities

**DOI:** 10.3390/ijms27209134

**Published:** 2026-10-15

**Authors:** Jieun Jeon, Hye Suk Baek, Kibeom Park, Mee-Na Park, Na Hyeon Heo, Sohyeon Cheon, Gahyeon Kim, Shin Kim

**Affiliations:** 1 Department of Immunology, School of Medicine, Keimyung University, Daegu 42601, Republic of Korea; 2 School of Medicine, Keimyung University, Daegu 42601, Republic of Korea

**Keywords:** colorectal cancer, T-cell exhaustion, immunometabolism, metabolic dysfunction, tumor microenvironment, immune checkpoint blockade, microbiota, therapeutic resistance, obesity, diabetes mellitus

## Abstract

Colorectal cancer (CRC) remains a major cause of cancer-related morbidity and mortality worldwide. Metabolic disorders such as obesity, diabetes mellitus, dyslipidemia, and metabolic syndrome are associated with CRC risk. Metabolic abnormalities may contribute to colorectal tumorigenesis and reshape the tumor immune microenvironment, which in turn influence immune dysfunction and therapeutic resistance. Exhausted CD8^+^ T cells exhibit impaired effector function, sustained inhibitory receptor expression, and transcriptional and epigenetic reprogramming. Metabolic stressors within the CRC microenvironment may modulate T-cell function and exhaustion-associated states. Additionally, other immune populations (natural killer cells, macrophages, myeloid-derived suppressor cells, dendritic cells, and regulatory T cells) may develop distinct dysfunctional or immunosuppressive states. Recent single-cell and spatial studies have indicated the heterogeneity of these immunometabolic interactions in CRC. This review examines the interactions of the systemic and tumor-associated metabolic alterations with T-cell exhaustion and other forms of immune dysfunction in CRC. We discuss the influence of glucose, lipid, amino acid, and mitochondrial metabolism, hypoxia, microbiota-derived metabolites, and chronic inflammation in shaping tumor–immune interactions. Finally, we reviewed therapeutic strategies targeting immunometabolic pathways and their potential integration with immune checkpoint blockade. Overall, understanding these interactions may help identify immunometabolic vulnerabilities for informing precise therapeutic strategies for CRC.

## 1. Introduction

Colorectal cancer (CRC) is one of the most prevalent malignancies worldwide. It remains a major cause of cancer-related morbidity and mortality despite substantial advances in screening, surgical techniques, chemotherapy, targeted therapy, and immunotherapy [1,2,3]. Historically, CRC incidence has been associated with aging populations and Westernized lifestyles; however, recent epidemiological studies have reported a rapid increase in CRC incidence in younger populations and in regions undergoing nutritional and socioeconomic transitions [4,5,6]. Additionally, metabolic disorders such as obesity, diabetes mellitus, dyslipidemia, and metabolic syndrome are found to be associated with CRC risk, and their global prevalence has increased substantially over recent decades [7,8,9].

Metabolic disorders influence CRC development and progression through multiple systemic and local mechanisms [10,11]. For example, obesity-associated chronic inflammation, insulin resistance, hyperglycemia, altered lipid metabolism, and gut microbiota dysbiosis may contribute to a tumor-promoting environment in the colon [12,13]. Compared with metabolically healthy individuals, patients with obesity or type 2 diabetes exhibit increased CRC incidence and poorer clinical outcomes [14,15]. Furthermore, metabolic syndrome has been associated with increased recurrence rates, enhanced metastatic potential, and reduced overall survival in patients with CRC [16,17]. These findings support an association between systemic metabolic dysfunction and CRC; however, the mechanisms linking systemic metabolic states to tumor and immune remodeling remain unclear [18].

Metabolic reprogramming is a defining characteristic of cancer cells, whereby malignant cells adapt to nutrient limitation, hypoxia, oxidative stress, and increased biosynthetic demands [19,20,21]. CRC cells exhibit extensive metabolic alterations such as enhanced aerobic glycolysis; dysregulated lipid metabolism, glutamine metabolism, and mitochondrial remodeling; and altered amino acid utilization [11,22,23]. The Warburg effect is a prominent feature of CRC metabolism. It is characterized by increased glucose uptake and lactate production even under normoxic conditions and contributes to the establishment of an acidic and nutrient-deprived tumor microenvironment (TME) [24]. CRC progression is frequently associated with hypoxia-inducible signaling, excessive reactive oxygen species (ROS) generation, and metabolic competition between tumor and immune cells [25,26]. These metabolic alterations support cancer cell proliferation and survival and may additionally influence antitumor immune responses [26,27].

Owing to recent advances in cancer immunology, T-cell exhaustion has been identified as a distinct state of T-cell dysfunction associated with persistent antigen stimulation in chronic disease and cancer [28,29]. In CRC, persistent antigen exposure drives T-cell exhaustion, while metabolic and other microenvironmental stresses modulate T-cell function and exhaustion-associated states [30,31,32,33]. T-cell exhaustion was initially characterized in chronic viral infections. Subsequently, exhaustion-associated T-cell states have been identified in solid tumors, including CRC [34,35]. Other immune populations, including natural killer (NK) cells, dendritic cells (DCs), macrophages, and other myeloid populations, may develop distinct dysfunctional or immunosuppressive phenotypes within the metabolically altered TME [36,37]. Tumor-associated macrophages (TAMs) frequently acquire immunosuppressive M2-like characteristics, whereas myeloid-derived suppressor cells (MDSCs) expand and inhibit T-cell activation through nutrient depletion and inhibitory cytokine production [38,39,40]. Regulatory T (Treg) cells further reinforce immune suppression through metabolic adaptation and cytokine-mediated inhibition, thereby contributing to an immunosuppressive environment that may facilitate tumor immune evasion and therapeutic resistance [41,42].

Metabolic alterations within the CRC TME may possibly contribute to multiple forms of immune dysfunction including impaired T-cell function. Within the TME, tumor cells compete with immune cells for glucose, amino acids, oxygen, and other nutrients. This competition may expose infiltrating immune cells to nutrient limitation, metabolic stress, mitochondrial dysfunction, and impaired bioenergetic fitness [43,44]. Lactate accumulation and extracellular acidification impair effector T-cell function and promote immunosuppressive macrophage phenotypes [45]. Lipid overload and oxidized fatty acids contribute to mitochondrial dysfunction and ferroptosis susceptibility in cytotoxic lymphocytes [46]. Amino acid depletion through enzymes such as indoleamine 2,3-dioxygenase 1 and arginase impair immune activation and promote tolerogenic immune states [47]. Hypoxia and adenosine signaling contribute to T-cell dysfunction and immune escape [48]. Thus, metabolic reprogramming influences multiple immune-cell states within the CRC TME, including pathways that modulate T-cell dysfunction [27,49].

The emerging field of immunometabolism deals with the relationship between metabolism and immunity and explores the regulation of immune cell differentiation, activation, and dysfunction by metabolic pathways [50,51]. In CRC, immunometabolic interactions are being increasingly recognized as potential contributors to tumor progression and therapeutic response. This concept is particularly relevant to microsatellite-stable/mismatch repair-proficient (MSS/pMMR) CRC, which accounts for the majority of CRC cases and generally responds poorly to immune checkpoint blockade (ICB). Although ICB has substantially improved outcomes in microsatellite instability-high/mismatch repair-deficient (MSI-H/dMMR) CRC, therapeutic resistance remains an issue as in MSS/pMMR disease. Therapeutic resistance has been associated with low immunogenicity, immune exclusion, stromal remodeling, and immunosuppressive features of the TME [52,53,54,55,56]. Therefore, whether metabolic interventions modify immune dysfunction and improve therapeutic responses in CRC should be investigated further.

Recent technological advances have enabled the characterization of spatial and functional heterogeneity of CRC immunometabolism [57,58,59,60]. Previous reviews have addressed metabolic reprogramming, immune dysfunction, and immunotherapy resistance in CRC from different perspectives [52,61,62]. In continuation, this review focuses on T-cell exhaustion in the context of metabolically dysregulated CRC. This perspective integrates host metabolic disorders and tumor metabolic reprogramming with CD8^+^ T-cell exhaustion and other distinct forms of immune dysfunction. Furthermore, it examines the contributions of these interconnected processes to TME remodeling and therapeutic resistance. Finally, therapeutic strategies targeting these immunometabolic pathways have been discussed.

## 2. Host Metabolic Disorders and CRC

CRC incidence has increased simultaneously with the global rise in metabolic disorders such as obesity, diabetes mellitus, dyslipidemia, and metabolic syndrome [63,64,65,66,67]. Increasing epidemiological evidence associates several metabolic disorders with increased CRC risk [9]. Concurrently, metabolic reprogramming within tumors has been implicated in cancer progression, metastatic dissemination, immune suppression, and therapeutic resistance [21,68,69,70]. Metabolic dysfunction induces chronic low-grade inflammation, hormonal dysregulation, oxidative stress, altered nutrient availability, and gut microbiota dysbiosis, all of which collectively establish a tumor-promoting environment in the colorectum [71,72,73,74] (Table 1). Systemic metabolic alterations additionally influence the CRC microenvironment by modifying nutrient availability, inflammatory signaling, and immune–stromal interactions [75,76,77]. Therefore, understanding the manner in which host metabolic disorders shape CRC development and the TME is important for defining the systemic origins of immunometabolic dysfunction.

### 2.1. Obesity and CRC

Obesity is one of the most important modifiable risk factors for CRC. According to epidemiological studies, increased body mass index, visceral adiposity, and waist circumference are associated with increased CRC incidence and mortality. Obesity-associated CRC risk is particularly prominent in individuals with central obesity, which suggests a critical role of visceral adipose tissue in colorectal carcinogenesis [78,95,96,97]. Currently, adipose tissue is recognized for its role in energy storage and as an active endocrine and immunological organ capable of secreting adipokines, inflammatory cytokines, growth factors, and metabolites that influence tumor biology [98,99,100].

Chronic low-grade inflammation is an important mechanism that links obesity to cancer development and progression [101,102]. In obesity, adipose tissue exhibits infiltration of macrophages, neutrophils, and other immune cells that produce pro-inflammatory cytokines such as tumor necrosis factor-α (TNF-α), interleukin (IL)-6, and IL-1β. Persistent inflammatory signaling activates oncogenic pathways including nuclear factor-kappa B (NF-κB), signal transducer and activator of transcription 3 (STAT3), and cyclooxygenase-2 (COX-2) pathways, which promote epithelial proliferation, angiogenesis, and tumor survival [103,104,105,106,107]. Furthermore, obesity-associated inflammation contributes to DNA damage and genomic instability through excessive ROS production and oxidative stress [108,109].

Adipokine imbalance further contributes to obesity-associated CRC progression. Leptin is a pro-inflammatory adipokine that is elevated in obesity. It promotes CRC cell proliferation, migration, angiogenesis, and stemness by activating the Janus kinase (JAK)/STAT, phosphoinositide 3-kinase (PI3K)/AKT, and mitogen-activated protein kinase (MAPK) pathways [79,110,111]. In contrast, adiponectin is an anti-inflammatory adipokine, typically exhibiting low levels in obesity. It exerts protective effects by improving insulin sensitivity and suppressing inflammatory signaling [112,113].

Obesity alters the metabolic milieu surrounding CRC. Circulating fatty acids and lipid metabolites at elevated levels promote lipid accumulation in tumor cells and infiltration of immune cells, thereby altering cellular metabolism and immune function [75,80]. Obesity-associated hypoxia in adipose tissue further exacerbates systemic inflammation and metabolic dysfunction through hypoxia-inducible factor-1α (HIF-1α)-mediated signaling [114,115]. According to preclinical CRC studies, obesity-associated metabolic alterations may influence therapeutic responses. In experimental CRC models, obesity-related conditions have been associated with 5-fluorouracil resistance and reduced efficacy of anti-programmed death-ligand 1 (PD-L1) therapy [81,116]. In patients with CRC, overweight and obesity conditions are associated with impaired T- and NK-cell cytotoxic function, whereas high-fat diet-induced obesity promoted polymorphonuclear MDSC accumulation in a murine CRC model [80,82].

### 2.2. Diabetes Mellitus and Hyperglycemia

Type 2 diabetes mellitus is another major metabolic disorder closely associated with CRC development and progression. Patients with diabetes exhibit increased CRC incidence, recurrence rates, and cancer-related mortality. Hyperglycemia, hyperinsulinemia, insulin resistance, and altered insulin-like growth factor signaling collectively contribute to colorectal tumorigenesis [13,117,118,119].

Chronic hyperglycemia promotes cancer progression through multiple mechanisms. Increased glucose availability enhances glycolytic metabolism and anabolic biosynthesis in CRC cells, thereby supporting rapid proliferation and survival [24,83]. Additionally, high-glucose conditions increase oxidative stress, which may contribute to cellular and DNA damage [120,121]. Hyperglycemia-induced advanced glycation end products and related receptor signaling may activate inflammatory and oncogenic pathways, including NF-κB and MAPK signaling pathways [83].

Hyperinsulinemia and increased insulin-like growth factor 1 (IGF-1) signaling are important in diabetes-associated CRC. Insulin and IGF-1 activate PI3K/AKT/mammalian target of rapamycin and RAS/RAF/MAPK pathways, which enhance cell proliferation, inhibit apoptosis, and promote angiogenesis. On persistent activation, these pathways contribute to tumor growth and metastatic progression [122,123]. Furthermore, insulin resistance often leads to compensatory hyperinsulinemia, thereby amplifying proliferative signaling within colorectal tissues [84,85].

Furthermore, diabetes-associated hyperglycemia reshapes antitumor immunity in CRC. In human CRC and experimental CRC models, hyperglycemia was associated with CD8^+^ T-cell dysfunction and immunosuppressive myeloid remodeling, including increased M2-like macrophage polarization and myeloid-cell accumulation [86,87]. Diabetic conditions reinforce systemic inflammation and tumor-promoting cytokine signaling [10,124]. Recently, diabetes-associated gut microbiota dysbiosis has been suggested to further contribute to CRC progression. For example, diabetes-associated gut microbiota dysbiosis has been linked to low-grade inflammation and metabolic dysfunction [125]. Independently, gut microbial dysbiosis disrupts intestinal barrier integrity and promotes mucosal inflammation, thereby creating conditions favorable for colorectal carcinogenesis [93]. Hyperglycemia-associated dysbiosis promotes intestinal barrier dysfunction, bacterial translocation, and chronic mucosal inflammation, which similarly create conditions favorable for colorectal carcinogenesis [12,125,126].

### 2.3. Dyslipidemia and Systemic Lipid Dysregulation

Dyslipidemia is characterized by elevated triglycerides, increased low-density lipoprotein (LDL) cholesterol, and reduced high-density lipoprotein cholesterol levels. It has been implicated in CRC development and progression [66,67].

Altered circulating lipid availability may influence colorectal tumorigenesis by changing the supply of fatty acids and cholesterol-derived metabolites to tumor and stromal compartments and by modifying inflammatory signaling within the TME. Cholesterol dysregulation is relevant to CRC because excessive cholesterol and its oxidized derivatives promote oxidative and inflammatory responses [75,88]. Oxidized LDL cholesterol activates macrophages and induces the production of pro-inflammatory cytokines such as TNF-α, thereby promoting tumor-supportive inflammation [89]. Therefore, these systemic lipid abnormalities may provide a metabolic context that favors tumor progression and shapes the lipid-rich microenvironment subsequently established through tumor-intrinsic metabolic reprogramming [75,127].

### 2.4. Chronic Inflammation and Metabolic Syndrome

Metabolic syndrome refers to a cluster of metabolic abnormalities including obesity, insulin resistance, hypertension, dyslipidemia, and hyperglycemia. Patients with metabolic syndrome exhibit an increased CRC risk and poor clinical outcomes [13,16,17]. Chronic inflammation is an important mechanism linking metabolic syndrome to colorectal carcinogenesis [13,71]. Persistent activation of inflammatory signaling pathways promotes epithelial proliferation, angiogenesis, stromal remodeling, and immune suppression [25,90]. Cytokines such as IL-6, TNF-α, and transforming growth factor-β (TGF-β) activate tumor-promoting signaling pathways such as JAK/STAT, NF-κB, MAPK, and PI3K/Akt pathways, thereby enhancing tumor growth and metastatic progression [72,90].

Inflammatory mediators influence metabolic programs within tumor and immune cells, thereby providing a potential link between systemic metabolic inflammation and local changes in the CRC microenvironment [25,128]. Chronic inflammation favors the recruitment and expansion of immunosuppressive myeloid populations such as MDSCs and M2-like TAMs through mechanisms such as STAT3-dependent MDSC expansion and C–C motif chemokine ligand 2–C–C chemokine receptor 2-mediated monocyte recruitment [91,92]. In experimental CRC models, obesity-associated chronic inflammation promoted MDSC generation and contributed to an immunosuppressive TME [82].

### 2.5. Gut Microbiota and Metabolic Inflammation

The gut microbiota is an important regulator of colorectal homeostasis, metabolism, and immunity. Alterations in microbial composition and function are commonly referred to as dysbiosis, and it is strongly associated with obesity, diabetes, and CRC [74,125]. The gut microbiota influences host metabolism through the production of short-chain fatty acids, bile acid metabolites, amino acid derivatives, and inflammatory mediators [12,74]. Beneficial microbial metabolites such as butyrate exhibit anti-inflammatory and antitumor properties that support epithelial integrity, regulate immune responses, and inhibit histone deacetylases [12]. In contrast, dysbiosis-associated metabolites such as trimethylamine-N-oxide, secondary bile acids, hydrogen sulfide, and N-nitroso compounds may promote inflammation, DNA damage, and tumor progression [12,74].

In experimental studies involving patients with CRC who showed immune suppression and chemoresistance, increased abundance of pathogenic bacteria such as *Fusobacterium nucleatum* was associated with poor prognosis. Microbiota-associated metabolic and inflammatory signals influence immune function within the CRC TME. Bacterial metabolites, such as short-chain fatty acids and polyamines, modulate T-cell differentiation, macrophage polarization, and DC activation [74,93]. Dysbiosis may promote the accumulation of immunomodulatory metabolites, including secondary bile acids, that can impair antitumor immune responses [74,94]. Recent studies additionally suggest that the microbiota may influence responsiveness to ICB. Across cancer types, bacterial communities such as *Akkermansia muciniphila*, *Bifidobacterium* spp., and *Faecalibacterium* spp. have been associated with improved antitumor immune responses and enhanced immunotherapy efficacy, whereas dysbiosis may be associated with reduced responsiveness to ICB [129,130]. However, CRC-specific clinical evidence remains limited.

## 3. Tumor Metabolic Reprogramming and Its Immune Consequences

**Figure 1:**
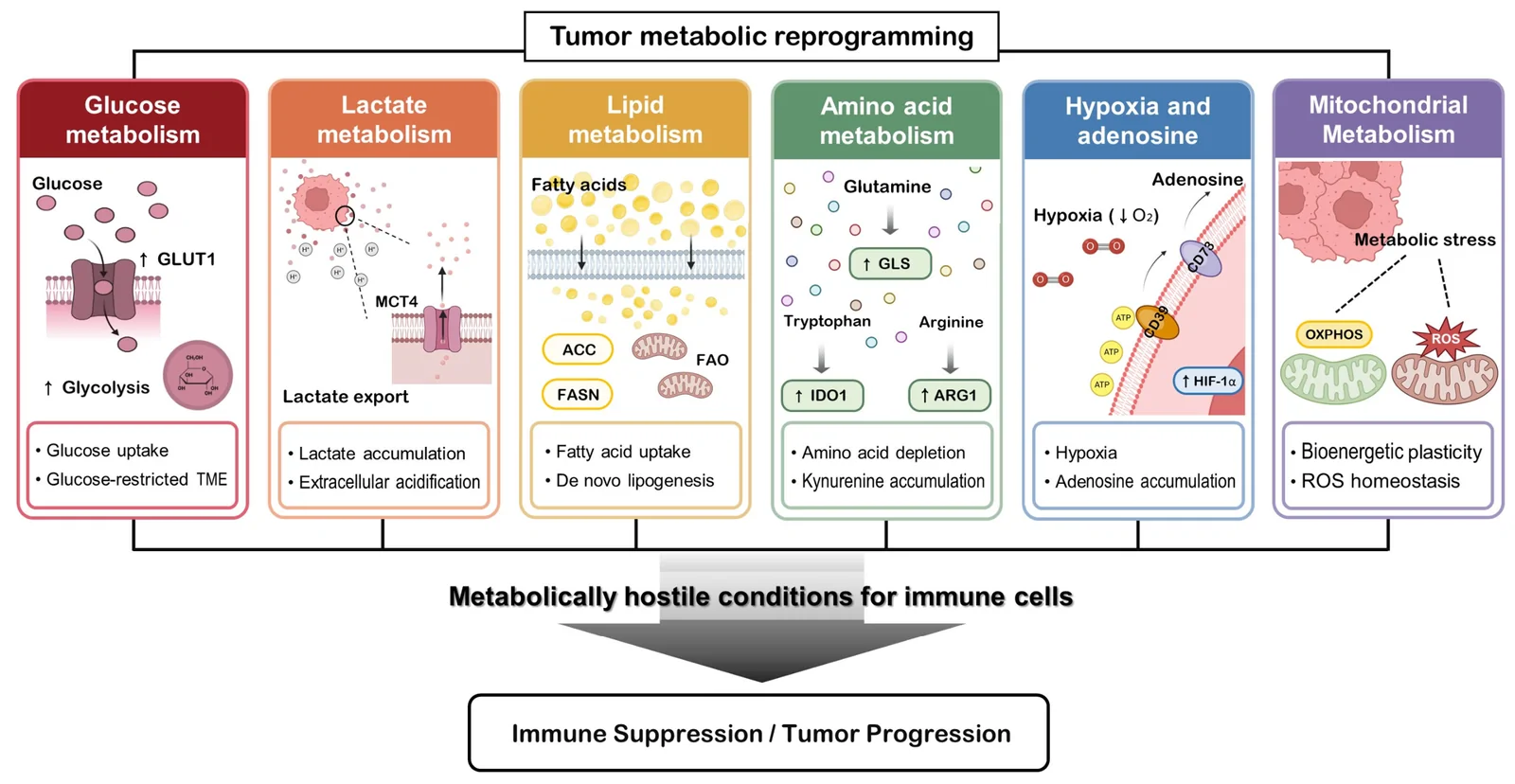
Tumor metabolic reprogramming and metabolically hostile conditions in the CRC TME. CRC cells undergo metabolic reprogramming involving glucose, lactate, lipid, amino acid, and mitochondrial metabolism, together with hypoxia- and adenosine-associated metabolic adaptations. Enhanced glucose uptake and glycolysis can contribute to a glucose-restricted tumor microenvironment, whereas lactate export and accumulation promote extracellular acidification. Lipid metabolic reprogramming involves fatty acid uptake, de novo lipogenesis, and fatty acid oxidation, while altered amino acid metabolism involves glutamine, tryptophan, and arginine pathways, contributing to amino acid depletion and kynurenine accumulation. Hypoxia is associated with HIF-1α stabilization and extracellular adenosine accumulation through CD39- and CD73-mediated ATP metabolism, whereas mitochondrial metabolic reprogramming is characterized by bioenergetic plasticity and altered ROS homeostasis. These metabolic alterations establish metabolically hostile conditions for immune cells and contribute to immune suppression and tumor progression within the CRC microenvironment. Upward and downward arrows in the text indicate increases and decreases, respectively. Created in BioRender. Kim, S. (2026) https://BioRender.com/bogqh1n. Abbreviations: ACC, acetyl-CoA carboxylase; ARG1, arginase 1; CRC, colorectal cancer; FAO, fatty acid oxidation; FASN, fatty acid synthase; GLS, glutaminase; GLUT1, glucose transporter 1; HIF-1α, hypoxia-inducible factor-1α; IDO1, indoleamine 2,3-dioxygenase 1; MCT4, monocarboxylate transporter 4; OXPHOS, oxidative phosphorylation; ROS, reactive oxygen species.

Metabolic reprogramming is a fundamental hallmark of cancer and an adaptive strategy that enables tumor cells to survive under conditions of nutrient limitation, oxidative stress, hypoxia, and immune surveillance [21,41,131]. In CRC, extensive alterations in glucose, lipid, amino acid, and mitochondrial metabolism support malignant progression while simultaneously reshaping the TME [27,49,73,132] (Figure 1 and Table 2). These metabolic adaptations sustain tumor proliferation and metastatic dissemination. They also establish metabolically hostile conditions for immune cells, which influences antitumor immunity [21,41]. In CRC, metabolic reprogramming contributes to immune dysfunction and immune escape [27,49,73,132].

Tumor cells continuously adapt their metabolic pathways in response to oncogenic signaling, environmental stress, nutrient availability, and interactions with stromal and immune populations [27,49,73]. Oncogenic mutations commonly observed in CRC include alterations in APC, KRAS, TP53, PIK3CA, and MYC. These influence metabolic phenotypes by regulating glycolysis, glutaminolysis, mitochondrial activity, and lipid biosynthesis [23,27,62,73]. Moreover, metabolic rewiring in CRC is closely associated with hypoxia, inflammation, and epigenetic remodeling, which contribute to the establishment of an immunosuppressive TME [49,62,73,127].

### 3.1. Glucose Metabolism and Lactate Signaling

The Warburg effect is the most prominent metabolic characteristic of CRC, involving enhanced aerobic glycolysis [24,27]. Rather than fully oxidizing pyruvate through mitochondrial oxidative phosphorylation, CRC cells preferentially convert glucose into lactate even in the presence of sufficient oxygen. This may be because, despite producing less ATP per molecule of glucose than oxidative phosphorylation, glycolysis enables rapid ATP generation and provides intermediates required for nucleotide, amino acid, and lipid biosynthesis, thereby supporting aggressive tumor growth [27,62,73,132].

In CRC, enhanced glycolysis is mediated by upregulation of glucose transporters and glycolytic enzymes. CRC tissues have shown increased expression of glucose transporter 1, hexokinase 2, phosphofructokinase, pyruvate kinase M2, and lactate dehydrogenase A in association with poor prognosis and metastatic progression [27,49,73,127,132]. Oncogenic KRAS and MYC signaling further amplify glycolytic activity by promoting transcription of glycolysis-related genes and increasing glucose uptake [23,27,73].

Glycolytic competition between tumor and immune cells has been implicated as an underlying mechanism that contributes to immune dysfunction across experimental tumor models. Activated effector T cells require glucose to support proliferation and cytokine synthesis. In CRC, high glycolytic activity may contribute to a glucose-restricted TME that limits nutrient availability to infiltrating immune cells [27,49,73,132]. Experimental studies have shown that reduced glucose availability disrupts T-cell metabolic programs and limits effector functions such as interferon-γ (IFN-γ) production [147].

A central consequence of glycolytic reprogramming is extracellular acidification driven by excessive lactate accumulation within the TME. CRC cells export lactate through monocarboxylate transporters (MCTs), particularly MCT1 and MCT4, leading to extracellular acidification [148,149,150]. Acidic TMEs facilitate epithelial–mesenchymal transition (EMT) and metastatic dissemination [149,150]. In CRC experimental models, tumor-derived lactate impaired CD8^+^ T-cell survival and effector function [133,134]. In CRC liver metastases, lactate-mediated acidification impairs NK-cell survival and antitumor function [135]. Lactate additionally impaired DC maturation in CRC models, which limited CD8^+^ T-cell responses [151]. By contrast, macrophages and Treg cells are relatively well adapted to lactate-rich environments [148,152].

### 3.2. Lipid Metabolic Reprogramming

Lipid metabolic remodeling contributes to CRC progression and immune dysfunction. CRC cells exhibit enhanced de novo lipogenesis, fatty acid uptake, cholesterol synthesis, and fatty acid oxidation. These enable CRC cells to adapt to metabolic stress and support their rapid proliferation [88,127,153]. CRC tissues show upregulation of fatty acid synthase (FASN), sterol regulatory element-binding proteins (SREBPs), and acetyl-CoA carboxylase (ACC), with frequent association with aggressive tumor behavior and poor prognosis [88,127,154]. Additionally, lipid metabolic reprogramming supports cellular membrane synthesis, lipid-derived signaling molecule production, and resistance to oxidative stress [88,127,155].

Fatty acid oxidation provides an alternative energy source under nutrient-limited conditions and contributes to metastatic adaptation. CRC cells with enhanced fatty acid oxidation better adapt to hypoxic and nutrient-limited conditions [88,127,136,137,156]. Dysregulated lipid metabolism impairs T-cell function, alters macrophage polarization, and disrupts DC antigen-presenting function [157,158,159]. Across tumor models, tumor-infiltrating CD8^+^ T cells accumulated in oxidized lipids through scavenger receptors such as CD36 [138,153,158,160]. CD36-mediated lipid uptake results in impaired cytotoxic activity because it promotes lipid peroxidation and increases susceptibility to mitochondrial dysfunction and ferroptosis [138,153,158,160]. In addition to affecting fatty acids, cholesterol metabolism influences immune cell function within the TME [127,154,158,160]. By contrast, immunosuppressive populations such as Treg cells and TAMs exhibit higher metabolic adaptability to lipid-rich environments [69,156,161].

### 3.3. Amino Acid Metabolism

CRC cells exhibit extensive alterations in amino acid metabolism to support biosynthesis, redox balance, and energy production [22,23,139]. Particularly, glutamine plays an important role in CRC metabolism. Glutamine acts as a carbon and nitrogen source for nucleotide synthesis, amino acid production, and tricarboxylic acid (TCA) cycle replenishment [23,27,139]. Many CRC cells display glutamine dependency and rely heavily on glutaminolysis for survival and proliferation. Glutamine metabolism is regulated by oncogenic pathways such as MYC and KRAS pathways. Increased expression of glutaminase, glutamine transporters, and TCA cycle enzymes has been associated with aggressive CRC phenotypes [22,23,139]. As glutamine is required for both tumor and immune cell functions, tumor-driven glutamine consumption may additionally restrict glutamine availability to infiltrating lymphocytes [139].

Altered tryptophan metabolism contributes to immune suppression in CRC. Indoleamine 2,3-dioxygenase 1 (IDO1) expression occurs in human CRC tissues, with high expression at the tumor invasion front associated with adverse clinical outcomes [140]. Simultaneously, MYC-driven tryptophan uptake and kynurenine-pathway metabolism have been observed in colon cancer, with higher kynurenine levels in human tumor tissues than in adjacent normal tissues [141]. According to experimental studies, tryptophan depletion activates the general control nonderepressible 2–eukaryotic translation initiation factor 2 alpha stress-response pathway in T cells, which suppresses protein translation and induces proliferative arrest [162]. In experimental CRC models, kynurenine activated aryl hydrocarbon receptor (AhR) signaling and promoted AhR-mediated FoxP3 transcription, which enhanced Treg differentiation and suppressed CD8^+^ T-cell responses [142].

Arginine metabolism contributes to immune dysfunction [163,164]. Generally, arginase 1 (ARG1) expressed by TAMs and MDSCs depletes extracellular arginine, which impairs T-cell receptor signaling and IFN-γ production [164,165]. Additionally, arginine deficiency reduces CD3ζ chain expression and impairs T-cell proliferation and responsiveness [163,164,165]. Thus, alterations in amino acid metabolism may contribute to nutrient competition and immune suppression within the CRC TME.

### 3.4. Hypoxia, Mitochondrial Adaptation, and Adenosine Signaling

Hypoxia is a common feature of rapidly growing CRC tissues and is a major driver of metabolic reprogramming [143,166]. Insufficient vascularization and excessive oxygen consumption create oxygen-deprived regions within tumors, which lead to the stabilization of HIF-1α. HIF-1α promotes metabolic adaptation by regulating glycolytic enzymes and glucose transporters [143,166,167,168]. Additionally, hypoxia suppresses mitochondrial oxidative phosphorylation and enhances lactate production, which contribute to extracellular acidification and immune suppression [23,168,169]. Mitochondrial metabolic reprogramming is another important component of metabolic adaptation in CRC. CRC cells exhibit substantial mitochondrial and bioenergetic plasticity, including variable reliance on oxidative phosphorylation and glycolysis to adapt to metabolic stress [73,144]. Dysregulation of mitochondrial function alters mitochondrial ROS homeostasis and associated signaling pathways, thereby contributing to CRC progression [73,145].

A major immunosuppressive consequence of hypoxia is extracellular adenosine accumulation [48,170]. Under stress conditions, extracellular ATP released from dying or activated cells is converted to adenosine by the ectonucleotidases CD39 and CD73 [170]. Adenosine suppresses immune responses by activating the adenosine A2A receptor (A2AR) and adenosine A2B receptor (A2BR) on T cells, NK cells, and myeloid populations [48,170]. In human CRC tissues, elevated CD73 expression is associated with tumor progression and poor prognosis, whereas in experimental CRC models, CD73-derived adenosine impaired CD8^+^ T-cell viability and effector function through A2AR signaling [146]. Adenosine signaling inhibits T-cell receptor activation, cytokine production, and cytotoxicity while promoting Treg differentiation and M2-like macrophage polarization [170].

## 4. Immune Dysfunction in CRC

**Figure 2:**
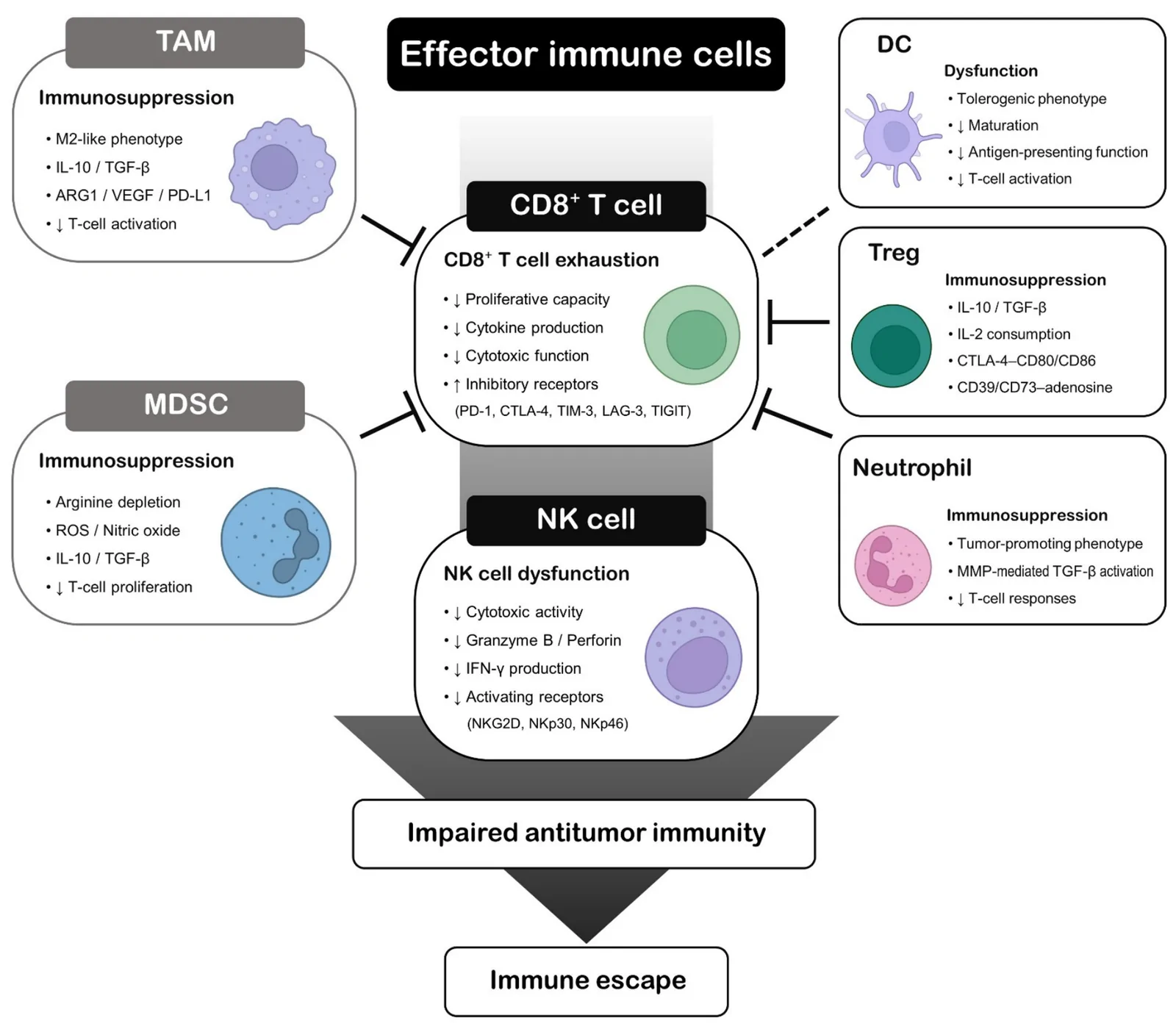
Immune cell dysfunction and immunosuppressive remodeling in the CRC TME. Immune dysfunction in CRC involves distinct functional alterations across adaptive and innate immune-cell populations. CD8^+^ T cells develop exhaustion characterized by impaired proliferative capacity, cytokine production, and cytotoxic function, whereas NK cells exhibit reduced cytotoxic activity and IFN-γ production. TAMs, MDSCs, Treg cells, and neutrophils contribute to immunosuppression and impaired T-cell responses through distinct mechanisms, while DC dysfunction is associated with impaired maturation, antigen-presenting function, and T-cell activation. These alterations contribute to impaired antitumor immunity and immune escape within the CRC tumor microenvironment. Upward and downward arrows in the text indicate increases and decreases, respectively. Blunt-ended lines indicate inhibition, whereas dashed lines indicate functional associations between immune-cell populations. Created in BioRender. Kim, S. (2026) https://BioRender.com/y2qgxu8. Abbreviations: CRC, colorectal cancer; TME, tumor microenvironment; TAM, tumor-associated macrophage; MDSC, myeloid-derived suppressor cell; Treg, regulatory T cell; DC, dendritic cell; NK, natural killer cell.

Immune dysfunction within the CRC TME contributes to impaired antitumor immunity and immune escape [28,29,35]. The progressive dysfunction of antitumor immune cells is collectively driven by persistent antigen exposure, chronic inflammation, metabolic stress, hypoxia, and immunosuppressive signaling within the TME [41,42,43,44,171]. T-cell exhaustion is characterized by impaired proliferative capacity, reduced cytokine production and cytotoxicity, altered metabolic fitness, and sustained expression of multiple inhibitory receptors [172,173,174]. T-cell exhaustion was initially characterized in chronic viral infections; however, it is currently well established in cancer including CRC [28,174]. In addition to T cells, other immune populations in CRC, including NK cells, DCs, macrophages, and MDSCs, acquire dysfunctional or immunosuppressive states [28,36,37] (Figure 2 and Table 3).

### 4.1. CD8^+^ T-Cell Exhaustion

CD8^+^ cytotoxic T lymphocytes are central mediators of antitumor immunity. They eliminate malignant cells through the secretion of effector cytokines such as IFN-γ and direct cytotoxic activity [174,199,200]. However, persistent antigen stimulation leads to T-cell exhaustion, which is a distinct dysfunctional differentiation state. Additionally, chronic inflammatory and metabolic stress further shape or reinforce exhaustion-associated states [174,201,202]. Unlike functional effector or memory T cells, exhausted T cells progressively lose proliferative capacity, cytokine production, and cytotoxic function, although they retain features of persistent activation [30,31,32,33,174]. Therefore, T-cell exhaustion is distinct from anergy, senescence, immune exclusion, and potentially reversible T-cell dysfunction caused by metabolic constraints within the TME. The latter includes biologically distinct mechanisms of impaired antitumor immunity [201,203]. A hallmark of T-cell exhaustion is the sustained co-expression of inhibitory receptors, including programmed cell death protein 1 (PD-1), cytotoxic T-lymphocyte-associated protein 4 (CTLA-4), T-cell immunoglobulin and mucin-domain containing-3 (TIM-3), lymphocyte activation gene-3 (LAG-3), and T-cell immunoreceptor with immunoglobulin and ITIM domains (TIGIT), with higher co-expression generally associated with more severe dysfunction and terminal exhaustion states [173,204,205].

Persistent tumor antigen exposure and chronic immunosuppressive signaling in CRC contribute to the development of CD8^+^ T-cell exhaustion, which impairs cytotoxic activity, cytokine production, and antitumor immune surveillance [29,206,207]. Functionally, exhausted CD8^+^ T cells exhibit impaired production of effector cytokines such as IFN-γ, TNF-α, and IL-2 with progressively diminished perforin- and granzyme-mediated cytotoxicity [33,56,173]. At the molecular level, exhaustion is characterized by extensive transcriptional, epigenetic, and metabolic remodeling. Additionally, metabolic alterations such as mitochondrial abnormalities, impaired glycolysis, oxidative stress, and reduced bioenergetic capacity are frequently observed in exhausted T cells [30,208,209].

T-cell exhaustion exists along a continuum and not as a single terminal state. Exhausted T cells comprise distinct differentiation states along this continuum. Progenitor exhausted T (Tpex) cells retain partial proliferative capacity and express T-cell factor 1 (TCF1), whereas terminal exhausted T cells exhibit severe dysfunction, high inhibitory receptor expression, and limited responsiveness to stimulation [202,204,210,211]. Tpex cells may respond to ICB, whereas terminal exhausted T cells are often epigenetically fixed and resistant to functional recovery [204,211,212].

Persistent glucose deprivation and related metabolic stress impair T-cell function and reinforce exhaustion-associated states [24]. Additionally, hypoxia may contribute to CD8^+^ T-cell dysfunction and exhaustion-associated inhibitory signaling [175]. In experimental CRC models, mitochondrial dysfunction was closely linked to CD8^+^ T-cell exhaustion, and perturbations in mitochondrial oxidative phosphorylation and mitochondrial homeostasis promoted CD8^+^ T-cell exhaustion [176,213].

### 4.2. Transcriptional and Epigenetic Regulation of T-Cell Exhaustion

T-cell exhaustion is shaped by coordinated transcriptional and epigenetic programs. For example, tumor-reactive CD8^+^ T cells from human CRC tissues showed distinct exhaustion-associated transcriptional and epigenetic states [177,178]. These cells exhibit an exhausted transcriptional signature and a distinct DNA methylation profile [177]. Exhaustion-associated transcription factors, including nuclear receptor subfamily 4 group A member 1 (NR4A1) and thymocyte selection-associated high mobility group box (TOX), have been functionally implicated in CRC. In an MC38 syngeneic colon tumor model, pharmacological NR4A1 antagonism reduced exhaustion-associated markers and enhanced effector function [179], whereas vascular endothelial growth factor A (VEGF-A) and IDO1-derived kynurenine promote TOX-associated T-cell exhaustion in CRC [180,181]. Furthermore, TOX silencing attenuates kynurenine-induced exhaustion and restores effector function in CD8^+^ T cells [180]. Epigenetic alterations, including changes in chromatin accessibility and DNA methylation, are associated with exhaustion-related states in CRC T cells [177,178,214].

### 4.3. NK-Cell Dysfunction

NK cells are critical components of innate antitumor immunity owing to their ability to eliminate transformed cells independent of antigen presentation [215,216]. However, NK-cell dysfunction is frequently observed in CRC, possibly contribute to impaired antitumor immunity [182,215,216]. Tumor-infiltrating NK cells in CRC commonly exhibit reduced cytotoxic activity, impaired IFN-γ production, and decreased expression of activating receptors such as NKG2D, NKp30, and NKp46 [216,217]. Simultaneously, increased expression of inhibitory receptors such as NKG2A and TIGIT further contributes to NK-cell dysfunction [183,217]. Dysfunctional NK cells exhibit impaired granzyme B and perforin release, which reduces their capacity to induce tumor-cell apoptosis [215,217].

Additionally, metabolic stress within the CRC TME contributes to NK-cell dysfunction [135]. In CRC, tumor glycolytic reprogramming, lactate accumulation, and hypoxia contribute to NK-cell metabolic and functional impairment [135,184,185]. TGF-β signaling further exacerbates NK-cell dysfunction within the CRC TME [185]. Mitochondrial dysfunction is another mechanism that contributes to NK-cell dysfunction in CRC [135]. In human colorectal liver metastases, lactate-mediated acidification reduced intracellular pH and promoted mitochondrial ROS accumulation in liver-resident NK cells, thereby increasing their susceptibility to apoptosis [135].

### 4.4. Myeloid Reprogramming and Immunosuppressive Remodeling

Myeloid populations play important roles in shaping the CRC immune landscape [36,37,40,92]. TAMs, MDSCs, neutrophils, and tolerogenic DCs collectively establish immunosuppressive environments that facilitate tumor progression and impair effector lymphocyte function [37,92]. TAMs are a dominant immune population in the CRC TME and frequently exhibit M2-like immunosuppressive phenotypes [36,37,92]. M2-polarized macrophages produce anti-inflammatory cytokines including IL-10 and TGF-β while expressing ARG1, VEGF, and PD-L1 [37,40]. These factors suppress T-cell activation, promote angiogenesis, and enhance metastatic dissemination [37,40,91].

Moreover, metabolic remodeling influences macrophage polarization. Lactate accumulation, hypoxia, and lipid metabolites favor M2-like macrophage polarization and suppress pro-inflammatory phenotypes [149,218,219]. In CRC, tumor-derived lactate promotes H3K18 histone lactylation in macrophages, thereby reprogramming them for tumor-promoting functions [186]. Additionally, MDSCs are frequently expanded in CRC, which contributes to profound immune suppression [165,187,188]. These cells inhibit T-cell proliferation through arginine depletion, ROS generation, nitric oxide production, and secretion of immunosuppressive cytokines such as IL-10 and TGF-β [165,188]. Furthermore, neutrophils may acquire tumor-promoting and immunosuppressive phenotypes within the CRC TME [189]. Tumor-infiltrating neutrophils suppress T-cell responses partly through matrix metalloproteinase (MMP)-mediated activation of TGF-β [190]. DCs in CRC frequently exhibit tolerogenic phenotypes characterized by impaired maturation and antigen-presenting function [191,192,193], which are induced by metabolic and tumor-derived signals, including hypoxia, lactate accumulation, and immunosuppressive cytokines. Ultimately, these signals promote impaired T-cell activation within the TME [191,192,193,194].

### 4.5. Treg Cells and Immune Suppression

Treg cells play important roles in maintaining immune tolerance; however, they are frequently co-opted by tumors to suppress antitumor immunity [41,42,148,152]. CRC tissues frequently exhibit increased infiltration of FoxP3^+^ Treg cells, although their prognostic significance remains context-dependent and may vary according to the composition of the tumor-infiltrating FOXP3^+^ T-cell populations [195,196]. Treg cells suppress effector immune responses through multiple mechanisms including IL-10 and TGF-β secretion, IL-2 consumption, CTLA-4-mediated inhibition of CD80/CD86-dependent costimulation, and metabolic competition [42,197,198]. Furthermore, Treg cells exhibit metabolic adaptability and may survive effectively within nutrient-deprived and lactate-rich TMEs that impair effector T-cell function [41,42,148,152,198]. Therefore, enhanced fatty acid oxidation and metabolic flexibility support Treg survival and suppressive function under metabolically stressful conditions, with fatty acid oxidation-derived acetyl-CoA promoting FoxP3 acetylation and stability [41,198]. Furthermore, Treg cells contribute to immune suppression through CD39/CD73-dependent adenosinergic signaling [170].

## 5. Stromal Remodeling and Immunometabolic Interactions in CRC

**Figure 3:**
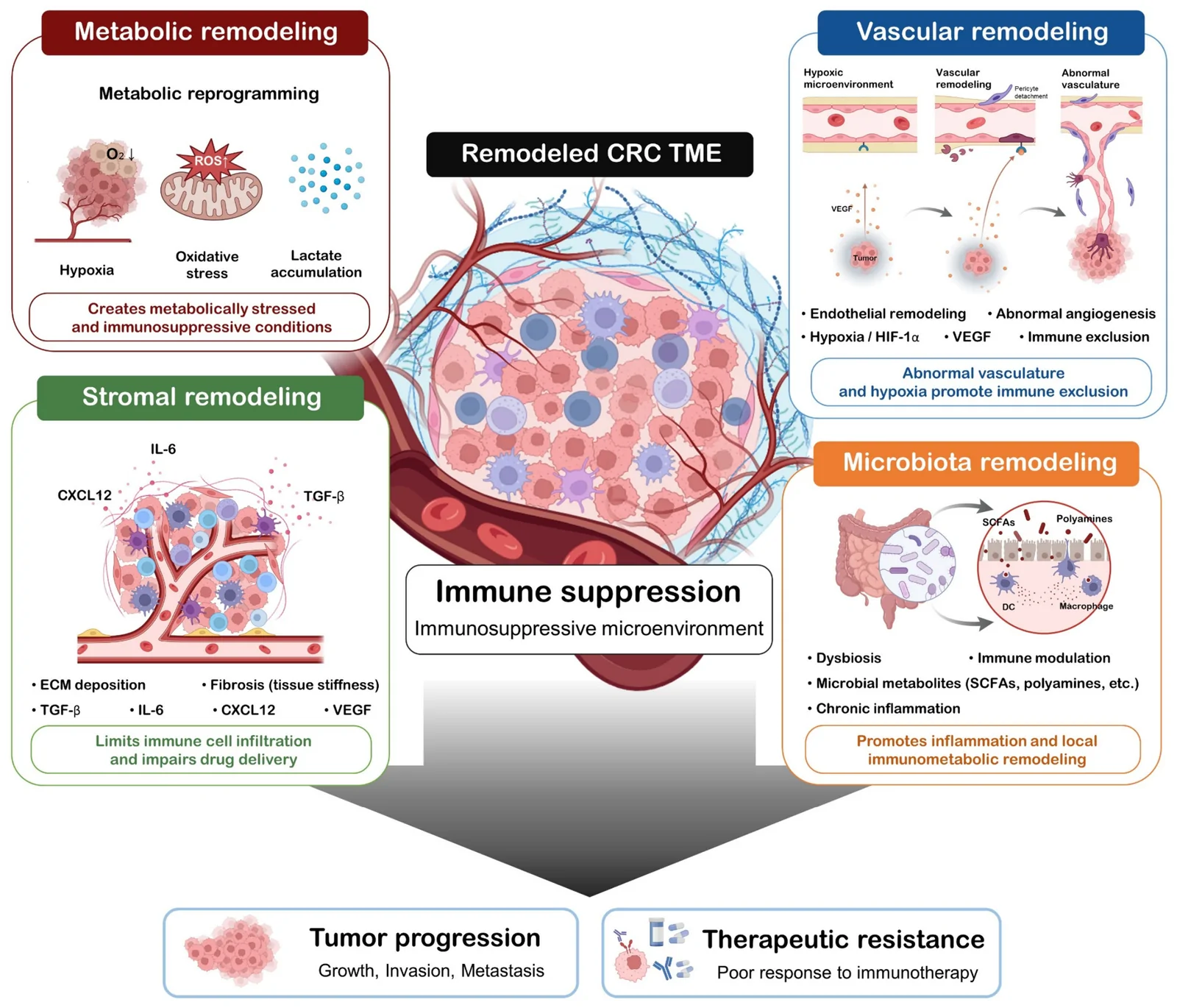
Stromal and microenvironmental remodeling in the CRC TME. Stromal and microenvironmental remodeling in CRC reflects interconnected metabolic, stromal, vascular, and microbiota-associated alterations within the TME. Hypoxia, oxidative stress, and lactate accumulation generate metabolically stressed and immunosuppressive conditions, while ECM deposition, fibrosis, and signaling through TGF-β, IL-6, CXCL12, and VEGF restrict immune-cell infiltration and impair drug delivery. Abnormal angiogenesis, persistent hypoxia, and endothelial remodeling promote immune exclusion, whereas microbiota dysbiosis and microbial metabolites influence inflammatory and immune responses within the CRC microenvironment. These interconnected alterations contribute to a remodeled CRC TME and an immunosuppressive microenvironment, promoting tumor progression and therapeutic resistance. Upward and downward arrows in the text indicate increases and decreases, respectively. Created in BioRender. Kim, S. (2026) https://BioRender.com/grfrtj2. Abbreviations: CRC, colorectal cancer; CXCL12, C-X-C motif chemokine ligand 12; DC, dendritic cell; ECM, extracellular matrix; HIF-1α, hypoxia-inducible factor-1α; IL-6, interleukin-6; ROS, reactive oxygen species; SCFA, short-chain fatty acid; TGF-β, transforming growth factor-β; TME, tumor microenvironment; VEGF, vascular endothelial growth factor.

The CRC TME comprises a dynamic and heterogeneous ecosystem of tumor cells, immune cells, stromal populations, extracellular matrix (ECM), vasculature, microbial components, and soluble mediators [25,36]. Metabolic dysregulation profoundly remodels this ecosystem to promote immune suppression, chronic inflammation, stromal activation, tissue fibrosis, angiogenesis, and therapeutic resistance [25,36,75] (Figure 3).

Furthermore, TME remodeling is not a passive consequence of tumor growth but an actively regulated process involving reciprocal interactions among malignant cells, stromal populations, and immune cells [24,75,220]. Within this network, stromal components, including cancer-associated fibroblasts (CAFs), the ECM, and tumor-associated vasculature, contribute to metabolic and immune remodeling of the TME through reciprocal interactions with tumor and immune cells [221,222,223,224,225]. Substantial spatial and functional heterogeneity has been observed within the CRC TME, which highlights the existence of metabolically specialized niches that influence local immune responses [58,75].

### 5.1. CAFs and Metabolic Crosstalk

CAFs are among the most abundant stromal populations in CRC, and they contribute to TME remodeling by actively regulating ECM deposition, cytokine secretion, metabolic adaptation, and immune responses within the TME [221,225]. Compared with normal fibroblasts, CAFs commonly exhibit activated phenotypes associated with markers such as alpha-smooth muscle actin and fibroblast activation protein; additionally, they enhance ECM remodeling [221,225]. Metabolic crosstalk between CRC cells and CAFs contributes to tumor progression [221,226]. CRC cells induce metabolic reprogramming in fibroblasts through tumor-derived signals and oxidative stress, which promote glycolysis and autophagy and facilitate lactate production and metabolic coupling that support tumor progression [226,227,228].

Additionally, CAFs contribute to immune suppression through the secretion of stromal signals such as TGF-β, IL-6, CXCL12, and VEGF, which impair cytotoxic T-cell infiltration and promote immune exclusion within the TME [221,222,225]. TGF-β signaling is particularly important for promoting immune exclusion and limiting T-cell recruitment in CRC [222,229,230]. Recent single-cell analyses have shown substantial heterogeneity among CAF populations in CRC, with distinct subsets exhibiting matrix-remodeling, myofibroblastic, vascular, immune-associated, or antigen-presenting phenotypes [225].

### 5.2. ECM Remodeling and Fibrosis

ECM remodeling is a hallmark of CRC progression that influences tumor and immune cell behavior within the TME [221,223]. ECM components including collagen, fibronectin, laminin, and hyaluronic acid are extensively reorganized during tumor progression through the activities of CAFs, TAMs, and tumor cells [221,223,224,225]. Excessive collagen deposition and tissue stiffening create physical barriers that limit immune cell infiltration and impair drug delivery [223,225]. Additionally, tissue stiffness alters mechanotransduction pathways in tumor and immune cells, thereby influencing cellular proliferation, migration, and immune function [223,231,232]. Furthermore, dense fibrotic regions are frequently associated with hypoxia, metabolic stress, and immune exclusion [222,223,225]. MMPs produced by CAFs and TAMs contribute to ECM remodeling and are associated with CRC progression and metastatic dissemination [221,223,231,233].

### 5.3. Angiogenic and Vascular Remodeling

Angiogenic and vascular remodeling within the CRC TME is closely linked to the development of hypoxic and metabolically stressed regions. Rapid tumor growth combined with abnormal vascularization creates oxygen-deprived regions that further promote angiogenic signaling and vascular remodeling [166,222]. HIF-1α activation promotes VEGF expression, which drives angiogenic responses and contributes to vascular remodeling within the TME [166]. However, tumor-associated vasculature is often structurally abnormal and functionally inefficient, which results in persistent hypoxia and impaired immune cell infiltration [166,222,234]. Abnormal vascular remodeling contributes to immune exclusion by limiting the infiltration of cytotoxic lymphocytes into tumor tissues [222,234,235]. Endothelial remodeling shapes an immunosuppressive vascular niche by altering immune cell recruitment and infiltration within the CRC TME [222,235].

### 5.4. Stromal–Immune Crosstalk and Immunosuppressive Niches

The functional states of infiltrating immune populations, including macrophages, are shaped by metabolic and stromal conditions within the CRC TME [219]. For example, TAM-derived cytokines and other mediators contribute to reciprocal interactions with stromal populations, particularly CAFs. These interactions promote immune suppression and tissue remodeling within the CRC TME [38,92,219,236]. In contrast, CAF-derived signals shape macrophage states and suppress T-cell responses to establish reciprocal stromal–immune regulatory circuits within CRC tissues [236,237]. In fact, interactions between TAMs and T cells contribute to the establishment and maintenance of immunosuppressive cellular niches within the CRC TME [219,236,238]. Moreover, TAM-derived cytokines such as IL-10 and TGF-β impair DC function and promote Treg cell induction, which further contribute to immune suppression [38,92].

In the CRC TME, MDSC accumulation and their functional states are shaped by inflammatory, tumor-derived, stromal, and metabolic signals [165,188]. Furthermore, interactions between MDSCs, TAMs, and Treg cells contribute to the formation of interconnected immunosuppressive cellular networks within the CRC TME [40]. Taken together, reciprocal interactions among stromal and immunosuppressive immune populations generate spatially organized niches that restrict effective antitumor T-cell responses [236,237].

### 5.5. Spatial Heterogeneity and Integrated Immunometabolic Remodeling

Recent technological advances have led to the identification of substantial spatial and cellular heterogeneity within the CRC TME [58,239]. Single-cell RNA sequencing, spatial transcriptomics, and multiplex imaging show that immune and stromal populations are spatially organized into distinct metabolic and functional niches that reflect the substantial variation in cellular composition, immune infiltration, and metabolic activity in different tumor regions [58,239,240,241]. Lactate-rich metabolic niches are associated with exhausted or suppressed T-cell states, immunosuppressive myeloid populations, and stromal remodeling within CRC tissues [242,243]. Additionally, cellular interactions within these niches form complex multicellular networks [58,239]. Furthermore, microbial metabolites contribute to the local immunometabolic environment by influencing immune-cell states and epithelial functions in CRC [74,93]. Cellular, stromal, and microbial components within these niches form interconnected signaling networks that shape local immune responses and therapeutic sensitivity [74,221,239]. ECM architecture and spatial organization influence immunotherapy responsiveness [60,232,244]. Overall, the spatial heterogeneity within the CRC TME reflects the integrated organization of tumor, stromal, and immune compartments into distinct microenvironmental niches [58,239,244]. Tumor metabolic reprogramming interacts with these stromal and immune alterations to create conditions that favor immunosuppressive populations while impairing cytotoxic antitumor responses [166,221,222]. Therefore, TME remodeling promotes tumor progression and establishes barriers to immunotherapy efficacy [126,223,245].

## 6. Therapeutic Resistance in Metabolically Dysregulated CRC

Immunotherapy has revolutionized cancer treatment by enabling durable antitumor immune responses in multiple malignancies [52,200]. In CRC, ICB targeting PD-1, PD-L1, and CTLA-4 has shown substantial efficacy in subsets of patients with MSI-H or dMMR tumors [52,246]. However, the majority of patients with CRC—particularly those with MSS or pMMR disease—exhibit limited responsiveness to current immunotherapies [52,56,60,246]. Therefore, resistance to immunotherapy remains a major clinical challenge in CRC treatment [52,58,207,246].

Metabolic dysregulation and T-cell exhaustion together with other forms of immune dysfunction contribute to immunotherapy resistance in CRC [62,76,211,247]. Tumor metabolic reprogramming and the resulting immunosuppressive TME collectively impair immune activation and limit responsiveness to checkpoint blockade [62,76,232,245,248].

T-cell exhaustion does not solely contribute to resistance to ICB. Human CRC studies indicate that MSS tumors may contain CD8^+^ T cells with predominantly tumor-unspecific or bystander-like features, which are indicative of limited tumor-specific T-cell activation [249]. Concurrently, functionally exhausted TIM-3^+^PD-1^+^ CD8^+^ T-cell populations have been directly identified in a subset of MSS CRCs [56]. Spatial profiling of MSS metastatic CRC further indicates that fibroblast-associated spatial organization restricts effective antitumor immune responses. This finding supports immune exclusion as an additional and biologically distinct resistance mechanism [60]. Immunotherapy resistance in CRC is highly heterogeneous and involves complex interactions between tumor-intrinsic, immune-mediated, metabolic, stromal, and microbial mechanisms [52,59,62].

### 6.1. Tumor Metabolic Reprogramming as a Barrier to Therapy

Tumor metabolic reprogramming establishes multiple barriers that limit the efficacy of ICB in CRC [61,76]. Although checkpoint blockade partially reinvigorates exhausted T cells, persistent metabolic stress within the TME constrains their functional recovery [61,250]. Rather than acting through a single metabolic pathway, several factors such as nutrient depletion, accumulation of immunosuppressive metabolites, hypoxia, and altered redox and mitochondrial homeostasis may collectively constrain immune-cell fitness and limit effective immune reinvigoration [61,76].

The strength of evidence is not equivalent between individual metabolic barriers in CRC. Various mechanisms that are commonly used to explain immunotherapy resistance, including nutrient competition, lactate accumulation, lipid metabolic stress, and hypoxia, are supported to varying degrees by pan-cancer or non-CRC experimental studies. Therefore, these should not be assumed to operate identically in human CRC [61,76,250]. By contrast, CRC-specific studies provide relatively direct evidence for particular metabolite-driven resistance mechanisms. In murine CRC models, intratumoral ammonia accumulation reprogramed T-cell metabolism, promoted T-cell exhaustion, and limited responsiveness to anti-PD-L1 therapy [251]. In patients with CRC, elevated serum ammonia and an ammonia-related gene signature were associated with altered T-cell responses, adverse outcomes, and lack of response to ICB [251].

A distinct nutrient-competition mechanism involved methionine and its downstream methyl-donor metabolism. Tumor-cell competition for methionine reduces intracellular methionine and S-adenosylmethionine (SAM) levels in CD8^+^ T cells, leading to the loss of H3K79me2, reduced STAT5 signaling, and impaired T-cell function [252]. Recent metabolomic profiling of human CRC tissues identified distinct SAM/S-adenosylhomocysteine (SAH) metabolism in MSS compared with that in MSI CRC. Additionally, experimental studies showed that colon cancer cells outcompeted CD8^+^ T cells for SAM and SAH; in an MSS CRC mouse model, SAM supplementation enhanced intratumoral CD8^+^ T-cell proliferation and reduced CD8^+^ T-cell exhaustion [253].

### 6.2. T-Cell Exhaustion and Other Forms of Immune Dysfunction

T-cell exhaustion is highly heterogeneous in CRC, and the differentiation states of exhausted T cells influence responsiveness to immunotherapy [29,56,211,254]. Exhausted T cells exist along a continuum ranging from progenitor-like populations with proliferative potential to terminally exhausted cells exhibiting substantial functional impairment and limited plasticity [211,220,254]. Tpex cells express TCF1 and retain partial responsiveness to checkpoint blockade. These cells proliferate and generate effector populations following PD-1 inhibition [211,254]. In contrast, terminal exhausted T cells exhibit high expression of PD-1, TIM-3, LAG-3, TIGIT, TOX, and NR4A transcription factors and frequently exhibit severe mitochondrial dysfunction and stable epigenetic programs [211,220,254]. In human CRC, this distinction is particularly relevant because MSS tumors contain tumor-unspecific or bystander CD8^+^ T-cell populations [249], whereas a subset of MSS CRC showed functionally exhausted TIM-3^+^PD-1^+^ CD8^+^ T cells [56].

In the metabolically dysregulated CRC TME, the development or maintenance of dysfunctional and exhausted T-cell states may be aided by chronic hypoxia, nutrient deprivation, oxidative stress, and inflammatory signaling [220,251,254]. These cells often fail to recover fully even after checkpoint blockade because exhaustion-associated epigenetic programs become fixed and resistant to reversal [211,220,254]. This incomplete reinvigoration is associated with distinct chromatin accessibility and transcriptional programs involving exhaustion-associated regulators such as TOX and NR4A [157,212]. Persistent antigen exposure combined with incomplete metabolic recovery may further sustain dysfunctional T-cell states following checkpoint blockade [211,254]. However, most of the mechanistic framework defining progenitor-to-terminal T-cell exhaustion and its epigenetic stabilization has been established in chronic infection or non-CRC tumor models. Therefore, these findings must be applied to human CRC with appropriate caution. Nevertheless, CRC-specific studies support substantial heterogeneity of intratumoral T-cell states and the presence of exhaustion-associated T-cell populations in human CRC [29,56].

Dysfunction in other immune lineages should be distinguished from T-cell exhaustion. In CRC liver metastases, lactate-mediated acidification impairs liver-resident NK-cell survival and function [135], which indicates that NK-cell dysfunction rather than T-cell exhaustion is the appropriate description of this phenotype. Similarly, MDSC expansion and Treg cell accumulation or metabolic adaptation are immunosuppressive remodeling processes that promote or maintain T-cell dysfunction, rather than the exhaustion of these populations [40].

### 6.3. Stromal and Myeloid Remodeling in Immune Exclusion and Therapeutic Resistance

Stromal remodeling and suppressive myeloid populations constitute major non-tumor-cell-mediated barriers to immunotherapy in CRC [40,221,255]. CAFs promote immune exclusion via ECM deposition and remodeling, which create physical barriers to T-cell infiltration, and through the secretion of immunosuppressive mediators such as TGF-β [221,224,255]. In human advanced CRC, high levels of fibrotic tumor components have been associated with altered intratumoral immune-cell infiltration. This finding provides CRC-specific evidence linking stromal remodeling to an immune-excluded tumor architecture [256]. These stromal barriers are particularly relevant to MSS/pMMR CRC, in which immune exclusion coexists with limited tumor-specific T-cell activation and dysfunction [56,60,249]. Spatial profiling of MSS metastatic CRC further identified fibroblast-associated spatial features linked to ineffective antitumor immune responses in the context of chemo-immunotherapy [60]. These findings support stromal organization as a barrier to effective antitumor immunity, although direct evidence that fibrosis itself causes resistance to ICB in human CRC remains limited.

TAMs and MDSCs suppress antitumor immunity through multiple immunosuppressive mechanisms [40,187,188]. ARG1, inducible nitric oxide synthase, ROS, IL-10, and PD-L1 collectively impair T-cell activation and promote T-cell dysfunction [40,187,188]. Consistent with the terminology used earlier, these changes indicate immunosuppressive myeloid remodeling rather than the exhaustion of TAMs or MDSCs. MDSC–Treg interactions may further reinforce immunosuppression in CRC [40], whereas lactate accumulation additionally amplifies stromal and myeloid immunosuppression, as discussed in earlier sections [148,219]. Thus, stromal remodeling, immune exclusion, and suppressive myeloid states limit effective antitumor immunity independent of and in combination with T-cell exhaustion.

### 6.4. Microbiota-Associated Modulation of Therapeutic Resistance

Microbiota-associated alterations may modulate therapeutic responses in CRC through effects on both tumor-cell stress responses and antitumor immunity. In patients with CRC, increased intratumoral *Fusobacterium nucleatum* abundance has been associated with recurrence following chemotherapy, whereas *F. nucleatum* promoted chemoresistance through TLR4–MYD88 signaling, microRNA regulation, and activation of autophagy [257]. Additionally, microbial metabolites may modulate responsiveness to ICB. In patients with metastatic CRC, non-response to immunotherapy was associated with greater *F. nucleatum* abundance and increased succinic acid levels. Mechanistically, *F. nucleatum*-derived succinic acid suppressed cGAS–interferon-β signaling, reduced CD8^+^ T-cell trafficking into the TME, and decreased sensitivity to anti-PD-1 therapy in experimental models [258].

Furthermore, microbiota-mediated resistance involves remodeling of immunosuppressive myeloid populations. In CRC mouse models, *Peptostreptococcus anaerobius* abolished the antitumor efficacy of anti-PD-1 therapy by promoting both the recruitment and immunosuppressive activity of MDSCs [259]. *P. anaerobius* activated integrin α2β1–NF-κB signaling in CRC cells to induce CXCL1-dependent recruitment of CXCR2^+^ MDSCs and directly enhanced MDSC suppressive activity through the bacterial protein lytC_22 and Slamf4 signaling [259]. These findings identify microbiota-associated signals as potential upstream regulators of myeloid remodeling that limit ICB in CRC.

However, microbiota-associated effects on therapeutic response appear to be context dependent rather than uniformly suppressive. In MSS CRC, high intratumoral *F. nucleatum* abundance was associated with a favorable response to anti-PD-1 therapy, and fecal microbiota transplantation (FMT) from patients with *F. nucleatum*-high tumors conferred anti-PD-1 sensitivity to germ-free mice [260]. Mechanistic experiments showed that *F. nucleatum*-derived butyrate inhibited HDAC3/8 in CD8^+^ T cells, increased TBX21 expression, reduced PD-1 expression, and enhanced effector function [260]. These apparently divergent effects of *F. nucleatum* indicate that microbiota-associated therapeutic responses may depend on microbial metabolic output, tumor context, and experimental conditions [258,260]. Thus, current evidence supports the microbiota as a potential modifier of therapeutic resistance in CRC rather than as a uniformly resistance-promoting factor. Hence, the predictive value of microbiota-associated features in patients requires further prospective validation.

### 6.5. Integrated Mechanisms of Therapeutic Resistance in CRC

The clinical efficacy of immune checkpoint inhibitors in CRC is strongly influenced by tumor molecular subtype [53,54]. MSI-H/dMMR tumors generally exhibit favorable responses to PD-1 blockade and are characterized by increased neoantigen generation and prominent immune infiltration [53,54,261,262]. Additionally, they frequently exhibit high densities of tumor-infiltrating lymphocytes, increased expression of immune checkpoint molecules, and inflamed TMEs [262]. Thus, the presence of inhibitory or dysfunctional T-cell states in MSI-H/dMMR CRC coexists with pre-existing antitumor immune activity that remains responsive to checkpoint blockade [53,54,262].

By contrast, MSS CRCs generally exhibit lower tumor mutational burden and reduced neoantigen burden, which limits effective T-cell priming and activation [55,261]. However, MSS/pMMR CRC should not be considered a uniform immunologically inactive state. As discussed, human CRC studies have indicated substantial heterogeneity within MSS disease, including tumor-unspecific or bystander CD8^+^ T-cell populations, functionally exhausted TIM-3^+^PD-1^+^ CD8^+^ T cells in a subset of tumors, and fibroblast-associated spatial barriers in metastatic disease [56,60,249]. Therefore, resistance in MSS/pMMR CRC cannot be attributed to T-cell exhaustion alone but may be a result of different combinations of insufficient tumor-specific T-cell activation, immune exclusion, T-cell dysfunction, and immunosuppressive metabolic and cellular conditions [56,60,249,251].

Furthermore, these resistance mechanisms interact and evolve during therapy. Therefore, the blockade of one inhibitory pathway may induce compensatory upregulation of alternative checkpoints including TIM-3, LAG-3, TIGIT, VISTA, and related suppressive pathways [263]. However, most of the mechanistic evidence for such adaptive checkpoint responses are derived from broader cancer models rather than CRC-specific clinical studies [263]. In CRC, persistent metabolic constraints, suppressive myeloid populations, and stromal barriers may act along with T-cell-intrinsic dysfunction to limit effective antitumor immunity [40,60,251]. Thus, therapeutic resistance in CRC reflects the convergence of tumor immunogenicity, T-cell differentiation states, metabolic fitness, stromal organization, microbiota-associated influences, and immunosuppressive cellular interactions rather than any single immune-dysfunction mechanism [56,60,251].

### 6.6. Precision Stratification of Immunometabolic Resistance

Identification of predictive biomarkers is essential for optimizing immunotherapy strategies in CRC. Established biomarkers such as MSI status provide important clinical information but do not fully capture the heterogeneity of therapeutic resistance in CRC [58,59]. Owing to this heterogeneity, patient stratification should move beyond individual metabolic or immune markers. In this context, integrated assessment of immune-cell states and their spatial organization may provide additional information on therapeutic response [58,59,60].

CRC-specific single-cell and spatial studies support this multidimensional approach. In MSS CRC, an immune-cell-related signature derived from single-cell and spatial transcriptomic analyses has shown association with clinical outcomes in patients receiving immunotherapy [58]. Spatiotemporal single-cell profiling has helped identify distinct cellular states and dynamics associated with differential responses to immunotherapy in patients with CRC [59]. Furthermore, human CRC studies have distinguished tumor-reactive from bystander CD8^+^ T-cell populations [249]. For example, distinct epigenetic features have been identified in tumor-reactive CD8^+^ T cells from patients with CRC [177]. These findings indicate that the functional identity of tumor-infiltrating T cells may reveal information beyond that provided by their abundance alone [177,249].

Metabolic and stromal features may provide additional layers of stratification. Ammonia-related signatures have been associated with adverse outcomes and a lack of response to ICB in CRC [251]. A CAF-related transcriptional signature has been associated with prognosis and immune characteristics in CRC. Its potential value for predicting immunotherapy response was evaluated computationally in CRC and further assessed using immunotherapy cohorts from other cancer types [225]. However, these findings did not establish prospective predictive utility in patients with CRC [225,251]. Hence, further prospective validation is required before these emerging biomarkers may be used to guide treatment selection in CRC.

## 7. Therapeutic Strategies Targeting Immunometabolic Dysfunction in CRC

The growing understanding of immunometabolic remodeling in CRC has led to increasing interest in therapeutic strategies aimed at alleviating immune dysfunction and restoring effective antitumor immunity [52,62,76,247]. A major reason for this focus is that despite conventional treatments such as surgery, chemotherapy, radiotherapy, and targeted therapy remaining important components of CRC management, durable therapeutic responses remain limited in many patients [52,55,61]. Although ICB has shown substantial efficacy in MSI-H CRC, most patients with MSS CRC exhibit primary or acquired resistance associated with metabolic dysfunction, suppressive TMEs, T-cell exhaustion, and other forms of immune dysfunction [52,53,54,61,76].

Recent advances in cancer immunometabolism have highlighted multiple therapeutic vulnerabilities involving glucose metabolism, lactate signaling, amino acid metabolism, lipid metabolism, mitochondrial dysfunction, hypoxia, stromal remodeling, and microbiota-associated inflammation [61,62,76] (Table 4). These pathways are highly interconnected, which suggests that combinatorial approaches targeting both immune checkpoints and metabolic mechanisms may provide therapeutic advantages compared with those of single-agent interventions [52,76,247]. These emerging therapeutic strategies aim not only to directly suppress tumor growth but also to improve the metabolic fitness and effector function of dysfunctional immune cells [76,211,247].

### 7.1. ICB and Combinatorial Immunotherapy

Immune checkpoint inhibitors targeting PD-1, PD-L1, and CTLA-4 constitute the most clinically advanced immunotherapeutic strategy in CRC [52,55,264]. Anti-PD-1 antibodies such as pembrolizumab and nivolumab have shown substantial efficacy in MSI-H/dMMR CRC [53,54,264]. Combination therapy with nivolumab and the anti-CTLA-4 antibody ipilimumab has expanded the clinical use of checkpoint blockade in MSI-H/dMMR CRC [264]. However, checkpoint blockade alone remains insufficient in most patients with MSS CRC because of therapeutic resistance-associated factors such as metabolic dysfunction and immune exclusion [55,77,263]. Hence, combinatorial immunotherapeutic strategies are being developed to overcome metabolic and immune resistance mechanisms [55,254,263].

In addition to established PD-1- and CTLA-4-directed therapies, dual or multiple checkpoint blockade targeting PD-1 together with TIM-3, LAG-3, TIGIT, or related inhibitory pathways is under active investigation [263,264]. These approaches aim to overcome compensatory inhibitory signaling and improve effector function [254,263,265]. Adoptive cellular therapies including CAR T cells, T-cell receptor-engineered T cells, and NK-cell therapies are additionally being explored in CRC [254,266]. However, the metabolically hostile CRC TME significantly impairs the persistence and function of transferred immune cells [264,266]. To address this challenge, strategies that enhance the metabolic fitness of engineered lymphocytes are being investigated as potential approaches to improve therapeutic efficacy [254,265].

### 7.2. Targeting Glycolysis and Lactate Metabolism

As enhanced glycolysis and lactate accumulation contribute to immunosuppressive remodeling in CRC, targeting tumor glycolytic metabolism is considered an effective therapeutic strategy [132,148,267]. Inhibition of glycolytic enzymes such as HK2, PKM2, and LDHA may reduce tumor growth while simultaneously alleviating metabolic immunosuppression within the TME [132,267]. LDHA inhibition has shown therapeutic potential in preclinical studies because it directly limited lactate production and extracellular acidification [132,152]. In a preclinical MC38 CRC model, pharmacological LDH inhibition with GNE-140 delayed tumor growth, increased glucose uptake by tumor-infiltrating T cells, and enhanced the antitumor activity of CTLA-4 blockade. However, LDH inhibitors such as GNE-140 have not yet advanced to clinical trials [284]. Reduced lactate accumulation may improve T-cell activation, cytokine production, and NK-cell cytotoxicity while limiting TAM polarization toward immunosuppressive phenotypes [148,250,267].

Other emerging therapeutic targets include the lactate transporters MCT1 and MCT4, which regulate lactate transport across the plasma membrane [152]. MCT inhibition may disrupt tumor metabolic adaptation and reduce lactate-mediated immune suppression [148,152]. Among MCT-targeting agents, the selective MCT1 inhibitor AZD3965 has been evaluated in a first-in-human phase I trial in patients with advanced cancers including a substantial subgroup with colorectal adenocarcinoma. The trial demonstrated pharmacodynamic target engagement and established a recommended phase II dose, although clinical antitumor activity was limited. MCT4 expression may contribute to resistance to selective MCT1 inhibition and potentially limit the efficacy of MCT1-selective therapeutic strategies [268]. Buffering extracellular acidity through pH-modulating therapies has additionally shown potential in preclinical settings [148,152]. However, complete inhibition of glycolysis may also impair activated immune cells, which rely on glycolytic metabolism for effector function [77,267]. Therefore, these therapeutic strategies must carefully balance suppression of tumor metabolism and preservation of immune metabolic fitness [132,267].

### 7.3. Targeting Amino Acid Metabolism

Amino acid metabolic pathways are major regulators of immune function in CRC [139]. Enzymes involved in tryptophan, arginine, and glutamine metabolism have been investigated as potential therapeutic targets [139]. IDO1 inhibitors are of substantial interest because IDO1-mediated tryptophan depletion promotes immune tolerance and T-cell dysfunction [139,180]. Preclinical studies have suggested that IDO1 inhibition may restore T-cell proliferation and reduce tryptophan-catabolism-mediated immune tolerance [142]. However, biological evidence supporting the IDO1–kynurenine pathway does not necessarily translate into therapeutic efficacy. The phase III ECHO-301/KEYNOTE-252 trial in advanced melanoma failed to show improvements in progression-free or overall survival with the IDO1 inhibitor epacadostat plus pembrolizumab versus pembrolizumab alone [269]. Although this trial was not conducted in CRC, it highlights the limitations of inferring therapeutic benefit solely from pathway-level or preclinical evidence. As arginine availability is critical for T-cell metabolic fitness and antitumor activity [163,164], therapeutic arginase inhibition may be a viable strategy. A first-in-human phase I study that included CRC cohorts evaluated the arginase 1/2 inhibitor INCB001158 as a monotherapy and in combination with pembrolizumab [270]. Although increased plasma arginine levels evidenced its pharmacodynamic activity, its antitumor activity was limited, and clear enhancement of intratumoral T-cell infiltration was not observed in CRC [270].

Targeting glutamine metabolism is another strategy that has been explored [22,139,271]. In CRC preclinical models, the glutamine antagonist JHU083 suppressed tumor growth and enhanced CD8^+^ T-cell-dependent antitumor immunity [271]. The glutaminase inhibitor CB-839 has been clinically evaluated in combination with capecitabine. In a phase I study that included patients with CRC, the combination was tolerated at biologically active doses; however, no objective responses were observed. Exploratory analyses have suggested that patients with PIK3CA-mutant CRC may derive greater benefit, pending further evaluation [272]. Nevertheless, immune cells also require glutamine for activation; hence, careful therapeutic optimization is essential [22,271,273]. Modulation of amino acid transporters and nutrient-sensing pathways such as mTOR and AMPK pathways has been proposed as another potential strategy to improve immune metabolic fitness and enhance immunotherapy responsiveness [273]. Overall, these findings emphasize that pharmacological reduction in a metabolite or inhibition of a metabolic enzyme should not be considered equivalent to restoration of antitumor immunity without direct functional immune endpoints.

### 7.4. Targeting Lipid Metabolism and Ferroptosis-Associated Pathways

Altered lipid metabolism is being increasingly recognized as an important contributor to immune dysfunction in CRC [75,127,153]. Excessive fatty acid accumulation, lipid peroxidation, and oxidative stress may impair cytotoxic lymphocyte function and contribute to the accumulation or activity of immunosuppressive immune populations [75,153]. Inhibition of FASN, ACC, and SREBPs has been investigated as a strategy to suppress lipid-dependent tumor growth in CRC [88,127,153]. A phase I window trial that included patients with resectable CRC has evaluated the FASN inhibitor TVB-2640 and yielded pharmacodynamic evidence of on-target inhibition of lipid metabolism [285]. Additionally, CD36-mediated lipid uptake has been investigated as a potential therapeutic target [131,153]. A preclinical study including the MC38 CRC model showed that targeting CD36 reduced oxidized lipid uptake and lipid peroxidation in tumor-infiltrating CD8^+^ T cells, thereby improving their effector function and tumor control [138].

Manipulation of ferroptosis pathways is another potential therapeutic strategy under investigation [46,274]. Although induction of ferroptosis in tumor cells may suppress tumor growth, excessive lipid peroxidation and disruption of antioxidant defenses such as the GSH–GPX4 pathway may increase T-cell susceptibility to ferroptosis, thereby impairing their survival and effector function [274]. Therefore, selective modulation of ferroptosis pathways has been proposed as a strategy to target tumor cells while preserving immune cell viability [274].

### 7.5. Targeting Mitochondrial Dysfunction and Oxidative Metabolism

Mitochondrial dysfunction is a prominent metabolic feature of exhausted T cells and is a potential therapeutic target for restoring antitumor T-cell function [73,275]. Preclinical CRC studies have shown that restoring mitochondrial fitness and oxidative metabolism improved antitumor T-cell function [275,276]. In the MC38 CRC model, forced expression of peroxisome proliferator-activated receptor gamma coactivator 1-alpha (PGC-1α) restored mitochondrial function, CD8^+^ T-cell activation, IFN-γ production, and antitumor immunity [276]. Similarly, deoxycholic acid (DCA) impaired mitochondrial fitness in CD8^+^ T cells by suppressing oxidative phosphorylation, increasing mitochondrial ROS, and inhibiting mitophagy, thereby promoting T-cell dysfunction and terminal exhaustion in CRC models [275].

Mitochondrial ROS signaling remains necessary for appropriate T-cell activation [277]. Pharmacological reactivation of mitophagy with Urolithin A restored CD8^+^ T-cell mitochondrial fitness and enhanced the antitumor efficacy of anti-PD-1 therapy in CRC models [275]. However, these mitochondrial-targeting approaches are supported primarily by preclinical intervention studies; hence, their therapeutic efficacy needs to be established in patients with CRC [275,276].

### 7.6. Targeting Hypoxia and Adenosine Signaling

As hypoxia-driven immune suppression contributes to therapeutic resistance in CRC, strategies targeting hypoxia-associated pathways and adenosine signaling are under active investigation [48,222,286]. Inhibition of HIF-1α may suppress glycolytic reprogramming, angiogenesis, and immunosuppressive remodeling [166,168]. Anti-angiogenic therapies targeting VEGF may normalize tumor vasculature and improve oxygen delivery, thereby reducing hypoxia-associated suppression [287,288]. Therefore, combining VEGF inhibition with ICB has been investigated as a strategy to enhance antitumor immunity [287,288]. However, the randomized phase II BACCI trial in refractory mCRC showed that the addition of atezolizumab to capecitabine and bevacizumab resulted in only a modest improvement in progression-free survival that was not considered clinically meaningful [289].

Extracellular adenosine signaling is another potential therapeutic target [170,286]. Inhibitors of CD39, CD73, and adenosine A2AR could reduce adenosine-mediated suppression of T and NK cells [48,170,286]. Hence, the anti-CD73 antibody oleclumab has been clinically evaluated in MSS mCRC. In the randomized phase Ib/II COLUMBIA-1 trial, the addition of oleclumab and durvalumab to FOLFOX plus bevacizumab produced a numerically higher objective response rate than that observed with standard therapy alone, but the efficacy results did not support further development of this combination [278]. Recently, the randomized phase II ARC-9 study evaluated the dual A2AR/A2BR antagonist etrumadenant in combination with zimberelimab, FOLFOX, and bevacizumab in previously treated mCRC. This regimen improved progression-free and overall survival versus those with regorafenib, although the contribution of adenosine receptor blockade could not be isolated from the effects of the combination regimen [279].

### 7.7. Targeting Gut Microbiota and Microbiota-Derived Metabolism

The association between the gut microbiota and therapeutic responsiveness in CRC has prompted the investigation of microbiota-directed strategies as potential adjuncts to immunotherapy. FMT is an approach to modify the intestinal microbial community. In CRC-bearing mice, FMT combined with anti-PD-1 therapy improved tumor control and survival compared with that observed with either treatment alone, with accompanying changes in gut microbial composition and circulating metabolites [280]. Consistent with the potential importance of donor microbial composition, FMT from responders with low *F. nucleatum* abundance conferred anti-PD-1 sensitivity to recipient mice, whereas transfer from *F. nucleatum*-high non-responders did not [258]. In the same experimental setting, depletion of intestinal *F. nucleatum* with metronidazole reduced circulating succinic acid levels and restored sensitivity to anti-PD-1 therapy, providing proof-of-concept that targeted manipulation of resistance-associated microbiota may modify treatment response [258].

Defined bacterial and probiotic approaches may provide relatively controlled alternatives to whole-community transplantation. In CRC mouse models, supplementation with *Lacticaseibacillus rhamnosus* Probio-M9 enhanced anti-PD-1-mediated tumor inhibition and promoted CD8^+^ T-cell infiltration and activation, with transfer experiments supporting a contribution from altered intestinal microbiota and metabolites [281]. *Bifidobacterium catenulatum* administration inhibited tumor growth across multiple CRC mouse models and enhanced anti-PD-1 efficacy in MSS CRC models by increasing intratumoral CD8^+^ T-cell infiltration [282]. Mechanistically, *B. catenulatum*-derived acetate activated CD8^+^ T cells through MCT4-dependent signaling. This provides a direct link between microbiota-derived metabolites and improved immunotherapy response [282]. These findings provide preclinical evidence that defined microbial interventions and microbiota-derived metabolites modulate antitumor immune responses and sensitivity to ICB in CRC [281,282].

However, microbiota-directed treatment in CRC remains predominantly supported by preclinical evidence. Differences in microbial composition, metabolite production, host diet, treatment exposure, and recipient microbial ecology may influence the effects of microbiota manipulation. Additionally, even the same bacterial species may produce divergent effects on therapeutic response under different conditions [258,260]. Hence, prospective clinical studies are needed to determine whether FMT, defined bacterial or probiotic interventions, and microbiota-derived metabolites may be safely and reproducibly integrated with established CRC therapies and to identify the patients most likely to benefit from these approaches.

## 8. Future Perspectives

The growing understanding of immunometabolic remodeling in CRC has substantially reshaped current concepts of tumor progression, immune dysfunction, and therapeutic resistance [57,58,59,60]. Accumulating evidence over the past decade has shown that metabolic dysfunction is not merely a secondary consequence of malignant transformation but may contribute to immune dysfunction and tumor evolution [21,69]. Nevertheless, multiple challenges remain in translating immunometabolic knowledge into clinically effective therapies for patients with CRC [52,55,61].

One of the most important future directions involves deeper characterization of the spatial and temporal heterogeneity of the TME [58,59]. CRC tissues contain highly heterogeneous metabolic and immune niches that differ substantially in oxygen availability, nutrient distribution, stromal composition, microbiota interactions, and immune infiltration [74,166,239]. Conventional bulk transcriptomic approaches do not adequately capture these complex cellular ecosystems [58,59,239]. However, recent advances in single-cell RNA sequencing, spatial transcriptomics, multiplex imaging, and metabolomics are beginning to identify the dynamic evolution of immune-cell states and metabolic dysfunction within distinct metabolic niches [58,59,239]. Hence, future studies that integrate multiomics technologies may identify previously unrecognized immune-cell states, spatial interactions, and metabolic vulnerabilities [58,59,239].

Another critical challenge involves the elucidation of the dynamic differentiation of exhausted T cells [211,254]. Emerging evidence suggests that exhaustion exists along a continuum rather than as a fixed terminal state [211,254]. Certain Tpex populations retain proliferative and functional potential, whereas terminally exhausted cells exhibit stable epigenetic programs and severe metabolic dysfunction [157,211,254]. How metabolic stress may influence the transition between these progenitor and terminal exhausted T cell states and whether these trajectories may be therapeutically redirected remain important unresolved questions [157,211,254].

Additionally, the interaction between metabolism and epigenetic regulation is an important area for future investigation [157]. Metabolic intermediates such as lactate, acetyl-CoA, α-ketoglutarate, and SAM directly influence chromatin remodeling and transcriptional programs [157,283,290]. Consequently, metabolic dysregulation may contribute to the stabilization of exhaustion-associated epigenetic states through alterations in histone modification and DNA methylation [157,283]. However, the extent to which metabolite-dependent epigenetic remodeling contributes to stable T-cell exhaustion in human CRC remains unclear.

The development of predictive biomarkers for immunometabolic dysfunction is another major priority [58,61,254]. Current biomarkers including MSI, tumor mutational burden, and PD-L1 expression provide limited predictive value for immunotherapy responsiveness in CRC [52,55,61]. Hence, comprehensive biomarker strategies that integrate metabolic signatures, exhaustion-associated transcriptional programs, microbiota composition, spatial immune architecture, and circulating metabolites are required for improving patient stratification and predicting therapeutic response [58,60,61,248]. Machine learning-based integrative models that combine histopathologic, transcriptomic, metabolomic, spatial, and microbiome data may help achieve these goals by improving the identification of immunometabolic states associated with therapeutic response [58,60,61,248].

Future therapeutic strategies may require combinatorial targeting of interconnected pathways [52,55,61]. Therapeutic resistance in CRC may involve interactions between metabolic stress, suppressive stromal remodeling, chronic inflammation, and inhibitory signaling; hence, single-agent approaches may be insufficient to overcome multiple resistance mechanisms [52,61,69,221]. Whether pharmacological correction of a metabolic abnormality is sufficient to restore durable antitumor immune function in patients with CRC remains unclear.

Suppressive myeloid populations and stromal remodeling may be relevant therapeutic targets [221,288]. In the context of microbiota-directed approaches, the important unresolved questions include how interindividual differences in microbial composition and metabolite production influence therapeutic responses and whether microbiome or metabolite profiles can help prospectively identify patients who are most likely to benefit [258,282].

Finally, future studies should increasingly emphasize longitudinal and systems-level analyses of immunometabolic evolution during tumor progression and treatment [59,60,61]. Immune and metabolic states within the TME are highly dynamic and continuously adapt in response to therapy [59,60,69]. Hence, real-time monitoring of these changes through liquid biopsy, metabolomic profiling, and longitudinal spatial analyses are necessary for developing adaptive therapeutic strategies [52,59,60,61]. Furthermore, longitudinal characterization of the interactions between metabolic dysfunction, immune-cell states, and TME remodeling may help inform precise and adaptive therapeutic strategies in CRC.

## 9. Conclusions

CRC is increasingly recognized as a metabolically and immunologically complex malignancy in which metabolic dysfunction may contribute to tumor progression, immune dysfunction, and therapeutic resistance [21,61,69]. Accumulating evidence suggests that systemic and microbiota-associated metabolic alterations may contribute to colorectal tumorigenesis and remodeling of the TME [9,69,74]. Persistent antigen exposure can drive CD8^+^ T-cell exhaustion, while metabolic stress within the TME may further modulate T-cell function and exhaustion-associated states [61,69,157]. T-cell exhaustion, NK-cell dysfunction, suppressive myeloid remodeling, and Treg-cell accumulation may represent distinct but interacting components of the immunosuppressive CRC TME [69,92,157,187].

Tumor metabolic reprogramming may alter the metabolic landscape of the TME and interact with immune dysfunction to sustain an immunosuppressive microenvironment [61,69]. Stromal remodeling, angiogenesis, and microbiota-associated signals may further contribute to the heterogeneous and dynamic nature of the CRC TME [74,221,239,288]. Although ICB has substantially improved outcomes for patients with MSI-H/dMMR CRC, therapeutic resistance remains common in MSS/pMMR disease [52,55]. Overall, these findings provide a rationale for investigating combinations of metabolic targeting and ICB, although their clinical benefit in CRC is yet to be established [52,61,288].

Hence, a deeper understanding of how metabolic alterations interact with distinct immune-cell states and the TME is warranted to identify immunometabolic vulnerabilities that could inform precise therapeutic strategies for CRC [52,61,69,288].

## 10. Literature Search Methodology

This narrative review was based primarily on original research and review articles published during the past 20 years identified through PubMed and Google Scholar. The topics covered in the literature related to colorectal cancer, metabolic dysfunction, obesity, diabetes mellitus, dyslipidemia, metabolic syndrome, tumor metabolic reprogramming, immunometabolism, CD8^+^ T-cell exhaustion, other forms of immune dysfunction, stromal remodeling, gut microbiota, and immune checkpoint blockade. The studies were considered in relation to the central framework of this review, which involved linking host metabolic dysfunction and tumor metabolic reprogramming to immunometabolic stress, T-cell exhaustion or other forms of immune dysfunction, and therapeutic resistance in CRC. When available, CRC-specific studies using human samples or experimental CRC models were prioritized. Evidence from broader cancer or immunological studies was retained for its relevance to general mechanistic concepts but was distinguished from CRC-specific evidence throughout the review.

## Figures and Tables

**Table 1:** Metabolic abnormalities associated with CRC and their potential impact on antitumor immunity.

Host Metabolic Factor	Major Metabolic Alterations	Representative Molecular Pathways	Potential Effects on CRC Biology	Potential Effects on Antitumor Immunity	Representative References
Obesity and visceral adiposity	Adipokine imbalance; elevated circulating fatty acids; chronic low-grade inflammation; adipose-tissue hypoxia	Leptin–JAK/STAT; PI3K/AKT; MAPK; NF-κB; STAT3; HIF-1α	Tumor-cell proliferation and survival; angiogenesis; oxidative stress; tumor progression	Impaired T- and NK-cell cytotoxicity; MDSC accumulation and immunosuppressive remodeling	[78,79,80,81,82]
Diabetes mellitus and hyperglycemia	Hyperglycemia; hyperinsulinemia; insulin resistance; altered IGF-1 signaling	AGE–RAGE; NF-κB; MAPK; PI3K/AKT/mTOR; RAS/RAF/MAPK	Enhanced glycolytic and anabolic metabolism; proliferation and survival; oxidative stress; tumor progression	CD8^+^ T-cell dysfunction; M2-like macrophage polarization; myeloid-cell accumulation and immunosuppressive remodeling	[83,84,85,86,87]
Dyslipidemia and systemic lipid dysregulation	Elevated triglycerides and LDL cholesterol; reduced HDL cholesterol; altered circulating fatty acids and cholesterol-derived metabolites	Lipid- and cholesterol-associated metabolic and inflammatory signaling	Altered lipid availability; oxidative and inflammatory responses; tumor-supportive metabolic conditions	Altered lipid availability to immune cells; pro-inflammatory macrophage responses	[67,75,88,89]
Metabolic syndrome and chronic inflammation	Combined obesity, insulin resistance, hyperglycemia, and dyslipidemia; persistent inflammatory signaling	NF-κB; JAK/STAT; MAPK; PI3K/AKT; CCL2–CCR2	Epithelial proliferation; angiogenesis; stromal remodeling; tumor growth and metastatic progression	Recruitment and expansion of immunosuppressive myeloid populations, including MDSCs and M2-like TAMs	[13,17,90,91,92]
Gut microbiota dysbiosis and altered microbial metabolism	Altered microbial composition; altered SCFAs, secondary bile acids, and other microbial metabolites	Microbial metabolite-mediated inflammatory and immune signaling	Barrier dysfunction; inflammation; DNA damage; tumor-promoting conditions	Altered T-cell, macrophage, and DC function; suppression of CD8^+^ T-cell effector responses	[74,93,94]

Host metabolic factors, including obesity, diabetes mellitus, dyslipidemia, metabolic syndrome, and gut microbiota dysbiosis, are associated with CRC development and progression through interconnected metabolic, endocrine, inflammatory, and microbial mechanisms. These alterations influence nutrient and lipid availability, inflammatory and oxidative signaling, and immune–stromal interactions, thereby shaping the metabolic and immune environment of CRC. Their effects on antitumor immunity include impaired lymphocyte function, immunosuppressive myeloid remodeling, and microbiota-associated modulation of immune responses, although the strength and type of supporting evidence varies across individual metabolic factors. Abbreviations: AGE, advanced glycation end product; AKT, protein kinase B; CCL2, C–C motif chemokine ligand 2; CCR2, C–C chemokine receptor 2; CRC, colorectal cancer; DC, dendritic cell; HDL, high-density lipoprotein; HIF-1α, hypoxia-inducible factor-1α; IGF-1, insulin-like growth factor 1; JAK, Janus kinase; LDL, low-density lipoprotein; MAPK, mitogen-activated protein kinase; MDSC, myeloid-derived suppressor cell; mTOR, mechanistic target of rapamycin; NF-κB, nuclear factor kappa B; NK, natural killer; PI3K, phosphoinositide 3-kinase; RAF, rapidly accelerated fibrosarcoma; RAGE, receptor for advanced glycation end products; RAS, rat sarcoma; SCFA, short-chain fatty acid; STAT3, signal transducer and activator of transcription 3; TAM, tumor-associated macrophage.

**Table 2:** Metabolic reprogramming in CRC and metabolic crosstalk with tumor immune microenvironment.

Metabolic Axis	Characteristic Alterations in CRC	Representative Regulators and Signaling Pathways	Metabolic Consequences	Effects on CRC Biology	Effects on Immune Cells and the TME	Representative References
Glucose metabolism and lactate signaling	Enhanced aerobic glycolysis and glucose uptake; increased lactate production	GLUT1; HK2; PFK; PKM2; LDHA; KRAS; MYC; MCT1/MCT4	Rapid ATP generation; biosynthetic intermediates; lactate accumulation; and extracellular acidification	Tumor growth; EMT; metastatic dissemination	Glucose restriction; impaired T-cell effector function; impaired CD8^+^ T-cell and NK-cell function	[24,133,134,135]
Lipid metabolism	Enhanced de novo lipogenesis; fatty-acid uptake; cholesterol synthesis; and FAO	FASN; SREBPs; ACC; CD36	Membrane synthesis; lipid-derived signaling; alternative energy production; lipid peroxidation	Proliferation; metabolic-stress adaptation; resistance to oxidative stress	Impaired T-cell function; altered macrophage and DC function; CD8^+^ T-cell lipid peroxidation and dysfunction	[127,136,137,138]
Amino acid metabolism	Glutamine dependency; altered tryptophan/kynurenine and arginine metabolism	GLS; MYC; KRAS; IDO1; GCN2–eIF2α; AhR; ARG1	TCA-cycle replenishment; tryptophan depletion and kynurenine accumulation; arginine depletion	Biosynthesis; redox balance; tumor-cell survival and proliferation	Restricted nutrient availability; impaired T-cell proliferation and effector function; Treg differentiation; suppressed CD8^+^ T-cell responses	[139,140,141,142]
Hypoxia, mitochondrial metabolism, and adenosine signaling	HIF-1α stabilization; mitochondrial and bioenergetic plasticity; extracellular adenosine accumulation	HIF-1α; CD39; CD73; A2AR	Enhanced glycolysis and lactate production; altered OXPHOS and mitochondrial ROS; adenosine accumulation	Metabolic adaptation; CRC progression	Immune suppression; impaired CD8^+^ T-cell viability and effector function; reduced T- and NK-cell activity	[143,144,145,146]

CRC cells undergo metabolic reprogramming involving glucose, lipid, amino acid, and mitochondrial metabolism, together with hypoxia- and adenosine-associated metabolic adaptations. These alterations support tumor growth, survival, and adaptation to metabolic stress while reshaping nutrient availability and metabolite accumulation within the tumor microenvironment. The resulting metabolic conditions impair antitumor immune-cell function, including T- and NK-cell activity, while contributing to immunosuppressive features of the CRC microenvironment. Abbreviations: ACC, acetyl-CoA carboxylase; AhR, aryl hydrocarbon receptor; ATP, adenosine triphosphate; A2AR, adenosine A2A receptor; ARG1, arginase 1; CRC, colorectal cancer; DC, dendritic cell; eIF2α, eukaryotic translation initiation factor 2 alpha; EMT, epithelial–mesenchymal transition; FAO, fatty-acid oxidation; FASN, fatty acid synthase; GCN2, general control nonderepressible 2; GLUT1, glucose transporter 1; GLS, glutaminase; HIF-1α, hypoxia-inducible factor-1α; HK2, hexokinase 2; IDO1, indoleamine 2,3-dioxygenase 1; KRAS, Kirsten rat sarcoma; LDHA, lactate dehydrogenase A; MCT, monocarboxylate transporter; MYC, myelocytomatosis; NK, natural killer; OXPHOS, oxidative phosphorylation; PFK, phosphofructokinase; PKM2, pyruvate kinase M2; ROS, reactive oxygen species; SREBP, sterol regulatory element-binding protein; TCA, tricarboxylic acid; TME, tumor microenvironment; Treg, regulatory T cell.

**Table 3:** Immune dysfunction and immunosuppressive mechanisms in the CRC microenvironment.

Immune Cell Type	Functional State	Metabolic and Microenvironmental Influences	Functional Impact	Evidence Context	Representative References
CD8^+^ T cells	T-cell exhaustion; impaired metabolic fitness	Persistent antigen stimulation; glucose deprivation; hypoxia; mitochondrial dysfunction; transcriptional and epigenetic remodeling	Reduced proliferative capacity, cytokine production, and cytotoxicity; sustained inhibitory receptor expression	Human CRC; experimental CRC models	[56,175,176,177,178,179,180,181]
NK cells	NK-cell dysfunction	Tumor glycolytic reprogramming; lactate-mediated acidification; hypoxia; mitochondrial dysfunction; TGF-β signaling	Reduced cytotoxic activity and IFN-γ production; impaired granzyme B and perforin release	Human CRC; murine CRC; CRC organoid models	[135,182,183,184,185]
Macrophages/TAMs	M2-like immunosuppressive phenotype; metabolic reprogramming	Lactate accumulation; hypoxia; lipid metabolites	Suppression of T-cell activation; production of immunosuppressive factors; tumor-promoting macrophage functions	CRC-specific and broader mechanistic evidence	[37,186]
MDSCs/neutrophils	Immunosuppressive myeloid activity	Arginine depletion; ROS; nitric oxide; immunosuppressive cytokines; TGF-β signaling	Inhibition of T-cell proliferation and responses	CRC-specific evidence	[165,187,188,189,190]
DCs	Tolerogenic phenotype; impaired maturation and antigen-presenting function	Hypoxia; lactate accumulation; immunosuppressive cytokines	Impaired antigen presentation and T-cell activation	Human CRC and broader mechanistic evidence	[191,192,193,194]
Treg cells	Immune suppression; metabolic adaptability	Nutrient deprivation; lactate-rich conditions; fatty acid oxidation; CD39/CD73-dependent adenosinergic signaling	Suppression of effector immune responses; maintenance of suppressive function under metabolic stress	Human CRC and broader mechanistic evidence	[41,42,170,195,196,197,198]

Immune dysfunction in CRC involves distinct alterations across adaptive and innate immune-cell types rather than a uniform exhaustion program. CD8^+^ T cells develop exhaustion, whereas NK cells exhibit functional impairment; myeloid cells acquire immunosuppressive phenotypes; DCs exhibit impaired maturation and antigen-presenting function; and Treg cells maintain immunosuppressive functions within the CRC microenvironment. Metabolic and microenvironmental factors, including nutrient deprivation, lactate accumulation, hypoxia, mitochondrial dysfunction, and immunosuppressive signaling, contribute to these alterations, with the supporting evidence ranging from human CRC studies and experimental CRC models to broader mechanistic studies. Abbreviations: CRC, colorectal cancer; DC, dendritic cell; IFN-γ, interferon-γ; MDSC, myeloid-derived suppressor cell; NK, natural killer; ROS, reactive oxygen species; TAM, tumor-associated macrophage; TGF-β, transforming growth factor-β; Treg, regulatory T cell.

**Table 4:** Therapeutic strategies targeting immunometabolic dysfunction in CRC.

Therapeutic Strategy	Representative Targets and Interventions	Proposed Mechanism	Potential Immunological Effect	CRC Evidence and Developmental Stage	Representative References
ICB and combinatorial immunotherapy	PD-1/PD-L1; CTLA-4; TIM-3; LAG-3; TIGIT; adoptive immune-cell therapies	Blockade of inhibitory signaling; enhancement of antitumor lymphocyte activity	Improved T-cell effector function and antitumor immunity	Established clinical use of PD-1/CTLA-4-directed therapy in MSI-H/dMMR CRC; other checkpoint combinations and cellular therapies currently under investigation	[53,54,55,264,265,266,267]
Glycolysis and lactate metabolism	LDH/LDHA; GNE-140; MCT1/MCT4; AZD3965	Reduced lactate production/transport and extracellular acidification	Improved T-cell metabolic fitness and effector function; reduced lactate-mediated immune suppression	Preclinical CRC (LDH inhibition); phase I advanced-cancer trial including CRC (MCT1 inhibition)	[268,269]
Amino acid metabolism	IDO1/epacadostat; ARG1/2/INCB001158; glutamine/JHU083; GLS/CB-839	Modulation of tryptophan, arginine, and glutamine metabolism	Potential restoration of T-cell metabolic fitness and antitumor function	Preclinical CRC and early-phase trials including CRC; negative phase III IDO1 trial in melanoma	[270,271,272,273]
Lipid metabolism and ferroptosis-associated pathways	FASN/TVB-2640; ACC; SREBPs; CD36; GSH–GPX4/ferroptosis pathways	Reduced lipid synthesis/uptake and modulation of lipid peroxidation	Reduced lipid-associated CD8^+^ T-cell dysfunction; preservation of immune-cell viability	Window trial in resectable CRC (FASN inhibition); preclinical CRC (CD36); ferroptosis strategies largely restricted to preclinical studies	[138,274,275]
Mitochondrial dysfunction and oxidative metabolism	PGC-1α; mitophagy; Urolithin A	Restoration of mitochondrial fitness, oxidative metabolism, and mitophagy	Improved CD8^+^ T-cell activation, IFN-γ production, and antitumor function	Preclinical CRC models	[276,277]
Hypoxia and adenosine signaling	HIF-1α; VEGF; CD39/CD73; A2AR/A2BR; bevacizumab; oleclumab; etrumadenant	Reduction in hypoxia- and adenosine-mediated immune suppression	Potential enhancement of T- and NK-cell activity and ICB responsiveness	Clinical studies in mCRC/MSS mCRC, including randomized phase Ib/II and phase II trials	[278,279,280]
Gut microbiota and microbiota-derived metabolism	FMT; *Fusobacterium nucleatum*/succinic acid; metronidazole; Probio-M9; *Bifidobacterium catenulatum*/acetate	Modulation of microbiota composition and resistance-associated microbial metabolites	Increased CD8^+^ T-cell infiltration/activation; improved anti-PD-1 responsiveness	Predominantly preclinical CRC evidence	[259,281,282,283]

Therapeutic strategies targeting immunometabolic dysfunction in CRC include ICB-based approaches and interventions targeting tumor and immune metabolism, hypoxia and adenosine signaling, and gut microbiota. These approaches aim to alleviate metabolically driven immune dysfunction and improve the metabolic fitness and antitumor function of immune cells while limiting tumor-supportive metabolic adaptations. However, the supporting evidence ranges from preclinical CRC models to clinical studies, and clinical efficacy remains unestablished for many approaches. Abbreviations: A2AR, adenosine A2A receptor; A2BR, adenosine A2B receptor; ACC, acetyl-CoA carboxylase; ARG1/2, arginase 1/2; CRC, colorectal cancer; CTLA-4, cytotoxic T-lymphocyte-associated protein 4; dMMR, deficient mismatch repair; FASN, fatty acid synthase; FMT, fecal microbiota transplantation; GLS, glutaminase; GPX4, glutathione peroxidase 4; GSH, glutathione; HIF-1α, hypoxia-inducible factor-1α; ICB, immune checkpoint blockade; IDO1, indoleamine 2,3-dioxygenase 1; IFN-γ, interferon-γ; LAG-3, lymphocyte activation gene 3; LDH, lactate dehydrogenase; LDHA, lactate dehydrogenase A; MCT, monocarboxylate transporter; mCRC, metastatic colorectal cancer; MSI-H, microsatellite instability-high; MSS, microsatellite-stable; NK, natural killer; PD-1, programmed cell death protein 1; PD-L1, programmed death-ligand 1; PGC-1α, peroxisome proliferator-activated receptor gamma coactivator 1-alpha; SREBP, sterol regulatory element-binding protein; TIGIT, T-cell immunoreceptor with immunoglobulin and ITIM domains; TIM-3, T-cell immunoglobulin and mucin-domain containing-3; VEGF, vascular endothelial growth factor.

## Data Availability

No new data were created or analyzed in this study. Data sharing is not applicable to this article.

## Abbreviations

The following abbreviations are used in this manuscript:

A2AR	adenosine A2A receptor
A2BR	adenosine A2B receptor
ACC	acetyl-CoA carboxylase
AGE	advanced glycation end product
AhR	aryl hydrocarbon receptor
AKT	protein kinase B
ARG1	arginase 1
ARG1/2	arginase 1/2
ATP	adenosine triphosphate
CAF	cancer-associated fibroblast
CCL2	C–C motif chemokine ligand 2
CCR2	C–C chemokine receptor 2
COX-2	cyclooxygenase-2
CRC	colorectal cancer
CTLA-4	cytotoxic T-lymphocyte-associated protein 4
DC	dendritic cell
DCA	deoxycholic acid
dMMR	deficient mismatch repair
ECM	extracellular matrix
eIF2α	eukaryotic translation initiation factor 2 alpha
EMT	epithelial–mesenchymal transition
FAO	fatty-acid oxidation
FASN	fatty acid synthase
FMT	fecal microbiota transplantation
GCN2	general control nonderepressible 2
GLS	glutaminase
GLUT1	glucose transporter 1
GPX4	glutathione peroxidase 4
GSH	glutathione
HDL	high-density lipoprotein
HIF-1α	hypoxia-inducible factor-1α
HK2	hexokinase 2
ICB	immune checkpoint blockade
IDO1	indoleamine 2,3-dioxygenase 1
IFN-γ	interferon-γ
IGF-1	insulin-like growth factor 1
IL	interleukin
JAK	Janus kinase
KRAS	Kirsten rat sarcoma
LAG-3	lymphocyte activation gene 3
LDH	lactate dehydrogenase
LDL	low-density lipoprotein
MAPK	mitogen-activated protein kinase
mCRC	metastatic colorectal cancer
MCT	monocarboxylate transporter
MDSC	myeloid-derived suppressor cell
MMP	matrix metalloproteinase
MSI-H/dMMR	microsatellite instability-high/mismatch repair-deficient
MSS/pMMR	microsatellite-stable/mismatch repair-proficient
mTOR	mechanistic target of rapamycin
MYC	myelocytomatosis
NF-κB	nuclear factor-kappa B
NK	natural killer
NR4A1	nuclear receptor subfamily 4 group A member 1
OXPHOS	oxidative phosphorylation
PD-1	programmed cell death protein 1
PD-L1	programmed death-ligand 1
PFK	phosphofructokinase
PGC-1α	peroxisome proliferator-activated receptor gamma coactivator 1-alpha
PI3K	phosphoinositide 3-kinase
PKM2	pyruvate kinase M2
RAF	rapidly accelerated fibrosarcoma
RAGE	receptor for advanced glycation end products
RAS	rat sarcoma
ROS	reactive oxygen species
SAH	S-adenosylhomocysteine
SAM	S-adenosylmethionine
SCFA	short-chain fatty acid
SREBP	sterol regulatory element-binding protein
STAT3	signal transducer and activator of transcription 3
TAM	tumor-associated macrophage
TCA	tricarboxylic acid
TCF1	T-cell factor 1
Tex	exhausted T cell
TGF-β	transforming growth factor-β
TIGIT	T-cell immunoreceptor with immunoglobulin and ITIM domains
TIM-3	T-cell immunoglobulin and mucin-domain containing-3
TME	tumor microenvironment
TNF-α	tumor necrosis factor-α
TOX	thymocyte selection-associated high mobility group box
Tpex	progenitor Tex
Treg	regulatory T-cell
VEGF	vascular endothelial growth factor
VEGF-A	vascular endothelial growth factor A
